# Tailored GAFF Parameters for Pentamethylcyclopentadienyl Rh(I/III) Complexes with α‑Diimine Ligands: Validation and Solvation Studies

**DOI:** 10.1021/acs.jpcb.5c07040

**Published:** 2025-12-23

**Authors:** Richard Jacobi, Konstantinos P. Zois, Alexander K. Mengele, Sven Rau, Leticia González

**Affiliations:** † Doctoral School in Chemistry (DoSChem), 27258 University of Vienna, Währinger Straße 42, 1090 Vienna, Austria; ‡ Institute of Theoretical Chemistry, Faculty of Chemistry, University of Vienna, Währinger Straße 17, 1090 Vienna, Austria; § Institute of Inorganic Chemistry I, 9189 Ulm University, Albert-Einstein-Allee 11, 89081 Ulm, Germany; ∥ Vienna Research Platform on Accelerating Photoreaction Discovery, University of Vienna, Währinger Straße 17, 1090 Vienna, Austria

## Abstract

Rhodium­(III) complexes of the general structure [(bpy)­Rh­(Cp*)­X]^*n*+^ (bpy = 2,2′-bipyridine, Cp* = pentamethylcyclopentadienyl, X = H_2_O, OH^–^, Cl^–^) are potent catalysts in a wide range of reactions, including NAD^+^ reduction and NADH oxidation, CO_2_ reduction, and hydrogenation of small organic molecules. Computational studies of these complexes require tailored force field parameters for accurate classical molecular dynamics. Here, we develop parameters compatible with the general Amber force field for [(bpy)­Rh^III^(Cp*)­Cl]^+^ and the reduced [(bpy)­Rh^I^(Cp*)], an important intermediate in many applications. As the general Amber force field is unable to describe the η^5^ hapticity of the Rh–Cp* bond, we implement a σ-bonded model. The force field is validated against density functional theory reference data including bond lengths, angles, dihedrals, and potential energy profiles. Finally, we demonstrate its applicability by analyzing the aqueous solvation environment around the Rh^III^ and Rh^I^ complexes, highlighting its utility for studying structure–environment interactions.

## Graphical abstract

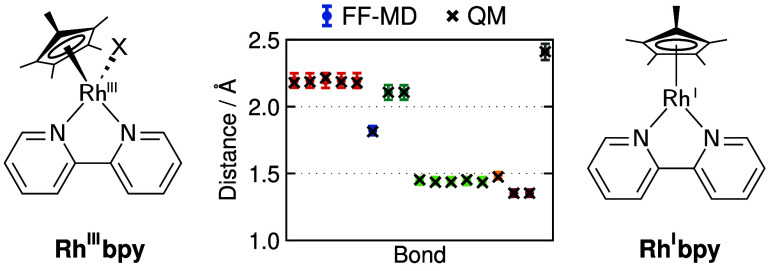

## Introduction

Rhodium­(III) complexes of the general structure [(bpy)­Rh­(Cp*)­X]^*n*+^ (bpy = 2,2′-bipyridine, Cp* = pentamethylcyclopentadienyl, X = H_2_O, OH^–^, Cl^–^, see Figure 1a) have emerged as versatile catalysts for a range of chemical, electrochemical, or photochemical applications. They have been successfully applied in NAD^+^ reduction,^1–25^ hydrogen production,^26–30^ CO_2_ reduction,^31–33^ and hydrogenation of unsaturated small organic molecules.^34^ Notably, these complexes can also catalyze reverse processes, for example, the oxidization of NADH to NAD^+^ in the presence of air, with concomitant generation of reactive oxygen species, which makes them potent anticancer agents.^35,36^ Integration of the catalytically active Rh^III^Cp* fragment into dyadic photocatalysts consisting of a photoactive metal center and bipyridine type bridging ligand has led to the development of highly active model systems for artificial photosynthesis with unparalleled catalytic activity.^37^ Detailed spectroscopic and time-dependent density functional theory characterization of such photocatalysts revealed important mechanistic insights into photodeactivation^25^ and thermal degradation pathways.^38^ In addition, anticancer activity has been reported for such Rh complexes when efficiently intercalated into DNA.^39,40^ This broad array of applications has generated the need for computational investigations of these complexes in their explicit environment. Such environments include solutions, the presence of catalysis products and educts, molecular assemblies, and the interaction of the complex within or at biopolymers, such as DNA.

**Figure 1:**
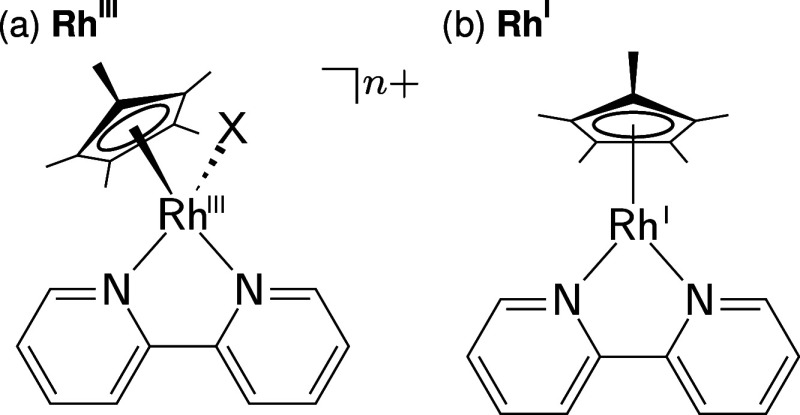
(a) [(bpy)­Rh^III^(Cp*)­X]^*n*+^ and (b) the corresponding reduced Rh^I^ form.

After 2-fold reduction and loss of the ligand X, [(bpy)­Rh^III^(Cp*)­X]^*n*+^ forms the neutral [(bpy)­Rh^I^(Cp*)] (see Figure 1b)—a crucial intermediate in many of these applications. This species is prone to oxidative addition of a proton, which is subsequently transferred along with the stored electrons, as a hydride to the substrate, which is finally reduced.^26,27,41^ Due to being prone to such proton addition, the interaction of [(bpy)­Rh^I^(Cp*)] with its surrounding environment is particularly interesting. As a result of a change in electrostatics upon reduction and ligand loss, these interactions are very different from those of the precatalytic form [(bpy)­Rh^III^(Cp*)­X]^*n*+^. Thus, at least these two forms of the complex represent important intermediates in catalysis, meaning that a computational model must be able to describe not one but two redox states. Moreover, as bpy can be substituted by other 2,2′-bipyridyl ligands—for instance, in order to immobilize the complex,^5^ improve DNA binding,^39,40^ or link the catalyst to a photosensitizer^25,30^ —generally applicable parameters are required to transfer to the broader family of Rh­(Cp*) complexes with different aromatic α-diimine ligands. Such applications, in which the interplay with the environment is critical,^42^ need models that explicitly account for these interactions.

Simulating metal complexes in an environment is challenging, because the metal center requires an accurate quantum mechanical description to capture its electronic structure correctly. However, applying the same high-level quantum treatment to the entire surrounding environment is computationally prohibitive, especially for larger systems or when many configurations must be sampled.^43–45^ To overcome such limitations, force fields are often employed. By approximating interatomic interactions with simple analytical functions, they offer a computationally efficient alternative, thereby enabling the simulation of large environments and supramolecular interactions at affordable cost.^43,46,47^ In practice, however, force field simulations require parametrization of analytical terms for each unique type of atom present in the system. While general-purpose force fields, such as the General AMBER Force Field,^48^ the CHARMM General Force Field,^49^ the OPLS,^50^ or the GROMOS force fields,^51^ are well suited for organic molecules, their applicability to transition metal complexes is limited. Modeling such systems requires custom parametrization of all relevant terms, extending well beyond the capabilities of standard libraries.

In this work, we present a force field parametrization for [(bpy)­Rh^III^(Cp*)­Cl]^+^ (Rh^III^-bpy) and [(bpy)­Rh^I^(Cp*)] (Rh^I^-bpy) designed to use with the General Amber Force Field (GAFF^48^ or GAFF2 ^52^). We show by comparing equilibrium bond lengths, angles, and dihedrals, and potential energy scans between the parametrized force field and a quantum mechanical reference that our parameters properly reproduce the geometry of the complex at both oxidation states. Moreover, we demonstrate that our parameters are applicable to α-diimine ligands other than bpy at the example of phen (1,10-phenanthroline) and dppz (dipyrido­[3,2-*a*:2′,3′-*c*]­phenazine). Finally, we demonstrate the relevance of our parameters by investigating the interactions between the complex and its environment using aqueous solvation shells as an example.

## Methodology

### General Considerations

The aim of this work is to develop fully covalently bonded models of Rh^I^-bpy and Rh^III^-bpy, which are transferable to analogous complexes with α-diimine ligands. We optimize force field parameters compatible with GAFF/GAFF2 to ensure compatibility with the Amber suite and take advantage of its optimized code and extensive analysis toolkit. All atomic interactions in GAFF/GAFF2 are modeled as a sum over bonds, angles, dihedrals, and nonbonded terms, the latter modeled as a 12-6 Lennard-Jones and a Coulomb potential:

1 $$E_{\mathrm{total}}=\sum_{\mathrm{bonds}}k_{b}{\left(r-r_{0}\right)}^{2}+\sum_{\mathrm{angles}}k_{\theta }{\left(\theta -\theta_{0}\right)}^{2}+\sum_{\mathrm{dihedrals}}V_{n}\left[1+\cos\left(n\phi -\gamma \right)\right]+\sum_{i=1}^{N-1}\sum_{j=i+1}^{N}[\frac{A_{\mathrm{ij}}}{R_{\mathrm{ij}}^{12}}-\frac{B_{\mathrm{ij}}}{R_{\mathrm{ij}}^{6}}+\frac{q_{i}q_{j}}{\epsilon R_{\mathrm{ij}}}]$$

where *r*, θ, and ϕ are bond lengths, bond angles, and dihedral angles; *r*_0_ and θ_0_ are equilibration structural parameters; *k*_b_, *k*_θ_, and *V*_*n*_ are force constants; *n* is the multiplicity and γ is the phase angle for torsional angle parameters; *R*_*ij*_ is the distance between atoms *i* and *j*; *A*_*ij*_ and *B*_*ij*_ are parameters characterizing the Lennard-Jones term; *q*_*i*_ and *q*_*j*_ are partial atomic charges; and ϵ is the dielectric constant. By default, all interactions in GAFF/GAFF2 are limited to point-to-point interactions of explicit atoms. However, the Cp*–metal interaction is an η^5^ bond, where each carbon contributes a fifth to the bonding. The archetypal example of this type of bonding is ferrocene, which contains two Cp (cyclopentadienyl) ligands rather than Cp*, and most of the approaches concerning η^5^ bonds in force fields, including those outlined in the following, are related to ferrocene. Translating η^5^ binding into a force field is not straightforward, and there are different approaches.^53^ In the rigid-body approach,^54^ bond lengths and angles are frozen, and thus cannot describe the flexibility of the complex. In the nonbonded approach,^55^ the bonded interactions are modeled purely electrostatically, which can lead to the dissociation of the complex. In the dummy-approach,^53,56,57^ a dummy atom (with no charge or mass) is placed at the center of the Cp* ring, and the ligand is bonded to the metal center by parametrizing the metal–dummy bond. However, within Amber,^58,59^ dummy atoms are currently included natively only as virtual sites, which cannot be used for bonded interactions. Nevertheless, as stated above, we want to ensure that the force field is fully compatible with the Amber suite. Thus, in this work, we employ the σ-bonding approach,^60^ where each metal–carbon bond is represented as a traditional bonded interaction. While conceptually straightforward, this approach does not properly capture the correct physics of the η^5^ bond. Therefore, particular care must be devoted to the parametrization to mimic the correct physics within this model. We ensure that our parameters for Rh^I^-bpy and Rh^III^-bpy preserve symmetry equivalencies in the complex as well as the free rotation of the Cp* ligand.

While the σ-bonding approach is conceptually simple and effective, it does not properly reflect the physical nature of a Rh–Cp* bond. Due to the bonding of the Rh to the electron cloud in the ring center, the Cp* ligand can freely rotate around this bond axis. As a result, the five aromatic carbon atoms in the Cp*–ligand are symmetry equivalent, which is a central property of the complex that needs to be reflected in the force field. This is not an issue when equilibrium bond lengths are assigned to the Rh–C bonds, as each of these bonds is of the same length by default. However, things get more complicated when assigning N–Rh–C (and Cl–Rh–C) equilibrium angles, which are paramount to adequately defining the overall ligand sphere around the metal center. In one single equilibrium geometry of the complex, there are different values for the N–Rh–C angles. This is shown exemplarily in Figure 2a, where, for instance, the purple N has a smaller N–Rh–C angle to the red C than to the green C at this frozen geometry. At the same time, the yellow N has a larger N–Rh–C angle to the red C, and a smaller to the green C. In total, there are 10 different N–Rh–C angles, which cluster into 5 symmetry-unique pairs in the optimized equilibrium structure, but they are not generally degenerate in every geometry. Still, the two nitrogens and five carbons need to be symmetry equivalent to properly capture the nature of the η^5^ bond. Assigning each of the N–Rh–C angles the equilibrium value they have at the optimized geometry would break the symmetry of the complex. To circumvent this issue, it is natural to assign averaged equilibrium angles, which thus effectively describe the angle between a N atom, the Rh center, and the center of the Cp* ligand. At any given conformation of the complex, there is a set of N–Rh–C angles with lower values than the assigned one, and a second set with higher ones. For instance, in the equilibrium geometry in Figure 2a, the angle including the purple N and the red C would be lower than the average, and the angle including the purple N and the green C would be larger than the assigned average. These terms will keep in balance, thus ensuring that the average angle is correct. However, on a more detailed level, there are two detrimental effects of this approach. First, each of the aromatic carbon atoms will be forced toward the center of the ring, thus nonphysically compressing the Cp* ligand. Second, the Cp* ligand (and in turn the other ligands) will be forced away from the Rh center, as larger distances to the Rh center allow for N–Rh–C angles which are closer to the averaged angle. These effects are an intrinsic consequence of the σ-bonding approach, when an η^5^-bond is approximated with five individual bonds, and there is no elegant conceptual way to prevent the distortions from happening. In order to counteract the effect that the Cp* ligand is compressed, the C–C equilibrium bond length parameters of the aromatic scaffold of Cp* can be increased compared to the equilibrium structure, and similarly, to counteract that the ligands are pulled away from the metal center, the Rh–C, Rh–N, and Rh–Cl equilibrium bond length parameters can be reduced. As such, the parameters are nonphysically altered from their actual values, but the physical geometry of the complex can be recovered. Counteracting the N–Rh–C angular terms in this way puts additional strain on adjacent parts of the complex, most notably the 5-membered ring formed by Rh and the bpy ligand, which requires additional parameter adjustment. As a consequence of these interdependent terms, we opted for an iterative process for the parametrization of equilibrium bond lengths and angles, where after each adjustment a short simulation is run and the bond lengths and angles are compared to target values until convergence is achieved. These target values are taken from the structure of the complex optimized using B3LYP^61–63^ /def2-SVP^64,65^ in Gaussian16 ^66^ (see details below).

**Figure 2:**
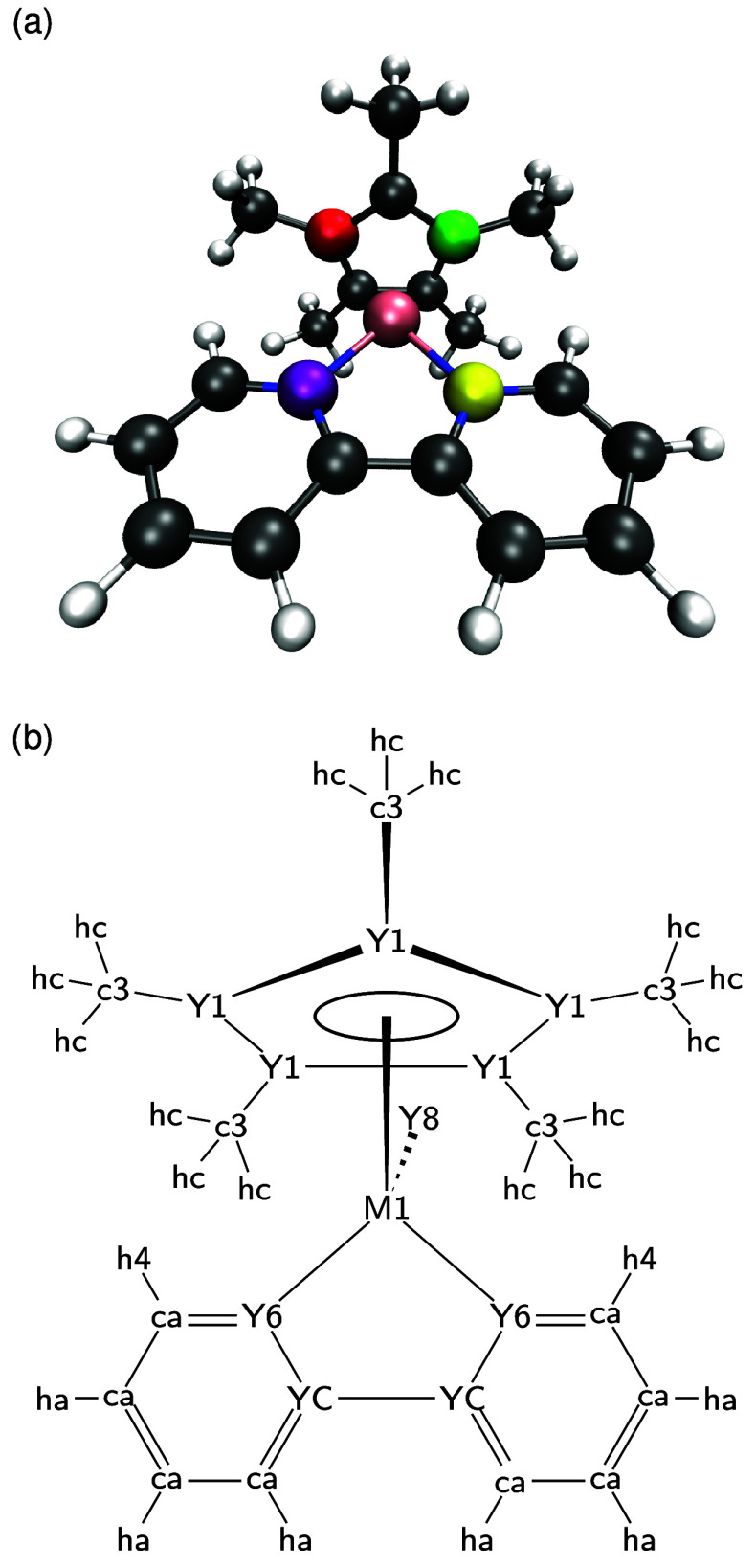
(a) Three-dimensional (3D) representation of Rh^I^-bpy, highlighting different N–Rh–C angles at the same geometry. (b) Atom types of the primitive complex Rh^I^-bpy and Rh^III^-bpy. Capital letters denote custom atom types, where M1 is Rh, Y1, and YC are C, Y6 is N and Y8 is Cl, while lowercase letters are standard GAFF2 atom types.

At this point, the classical bonded terms of the bonds including Rh lose their physical meaning to some degree and are rather used to mimic the physical behavior of the metal center. In some sense, this is similar to force fields having less physical meaning than wave functions, but they can nevertheless describe the behavior of molecules to a reasonable extent in the first place. To ensure that the terms still capture the geometry of the complex, they need to be adjusted not only to describe the equilibrium geometry but also capture dynamic effects. Thus, we compare the energy profiles resulting from the force fields with the quantum mechanical geometry scans.

### Reference Data

To validate the force field parameters, average bond lengths, angles and dihedrals in the simulations are compared to target values taken from the structure of the complex optimized using B3LYP^61–63^ /def2-SVP^64,65^ in Gaussian16.^66^ Dispersion effects are corrected for empirically using Grimme’s D3 model with Becke–Johnson damping,^67^ and implicit solvent effects for water are modeled using a conductor-like polarizable continuum model.^68,69^ Convergence of the geometry optimization is verified by the absence of imaginary frequencies exceeding 10 cm^–1^.

Energy scans performed with the force field are compared to energies computed on the same geometries with the more accurate double-hybrid B2PLYP^70^ functional with the ORCA6 package (version 6.0.1),^71–73^ where the domain-based local pair natural orbital (DLPNO)^74,75^ method with tight cutoff criteria (tightPNO settings^76^) is used to speed up the MP2 part of the calculation. For nonmetal atoms, the ZORA-def2-SVP^64,65,77^ basis is used, while the SARC-ZORA-TZVP basis set is used for Rh. Disperison corrections are included using Grimme’s D4 model,^78–81^ relativistic corrections via the zeroth order regular approximation (ZORA),^77^ and implicit solvent effects in water with the conductor-like polarizable model.^82^ For better convergence, the tightSCF keyword is used.^83^ The RIJCOSX approximation^84–90^ is used together with the SARC/J auxiliary basis set, while for the correlated DLPNO-B2PLYP method, the auxiliary basis is constructed automatically in ORCA using the AutoAux keyword.

### Parametrization Procedure

The parameters are optimized for Rh^I^-bpy and Rh^III^-bpy, but they are applicable to a range of α-diimine ligands, as shown below for phen and dppz. The parametrization procedure is as follows. To properly capture the electrostatics of the complex, restricted electrostatic potential (RESP) charges are fitted with the antechamber program included in Ambertools on the electrostatic potential computed with B3LYP/def2-SVP. As this electrostatic potential is computed on a single, optimized geometry, which does not capture symmetry effects due to rotations, slightly different charges are assigned for symmetry equivalent atoms. Consequently, the charges are symmetrized by averaging, thus enforcing the 5-fold rotational symmetry of the Cp* ligand as well as the vertical symmetry axis in the bpy ligand. Initial GAFF2 parameters for the Rh center are generated with the MCPB.py application included in Ambertools.^58,59^ In the MCPB workflow, force constants for bonds and angles are generated from a frequency calculation performed with B3LYP/def2-SVP as described above. For this, custom atom types are automatically assigned for the Rh center (M1), the aromatic carbon atoms in the Cp* ligand (Y1 through Y5), the metal-binding nitrogen atoms (Y6 and Y7), and the chloride ligand (Y8, see Figure 2b). To enforce symmetry equivalencies in the complex, Y1 through Y5 are all assigned Y1, and Y7 is assigned Y6, and in order to explicitly control the geometry of the 5-membered ring formed by the Rh center and the bpy ligand, the 2 and 2′ carbons are assigned the custom atom type YC. For the atom types relating to atoms included in GAFF2, i.e., Y1, Y6, Y8, and YC, the atomic mass, van der Waals radius, and the 12-6 potential well depth are assigned the same values as the corresponding GAFF2 standard atom type, i.e., ca, nb, cl, and ca, respectively. We also transfer the parameters for the atomic polarizability to our force field modification files, even though they are not used in our nonpolarizable simulations. All other atoms are assigned the default GAFF2 atom types according to Figure 2b. Whenever MCPB.py assigns different parameters for otherwise equivalent terms due to how the initial parametrization is performed on the optimized geometry, both force constants and equilibrium values are averaged between the respective terms. The resulting force constants are not altered in the parametrization process in order to stay closest to the results of the frequency calculation, with the exception of the N–Rh–C, N–Rh–Cl, and C–Rh–Cl angle terms, as the comparison of the energy profiles (see below) reveals that MCPB vastly overestimates those. Other than this, only the equilibrium values are adapted to ensure that the average bond lengths and angles during unconstrained molecular dynamics (MD) simulations agree with the B3LYP/def2-SVP optimized reference geometry (see below). We conclude the iterative process once the error is below 0.01 Å for all bond lengths and below 1° for all angles. The final parameters are presented in Tables 1 and 2. In addition to the parameters listed, each nonstandard atom type excluding M1 requires a set of parameters which is set equal to the corresponding parameters in GAFF2. For instance, the parameters for the Y1–Y1–c3 angle are identical with those of ca–ca–c3 in GAFF2. All force constants for the dihedrals, which include M1 are set to 0, as no constraints in addition to the bonds and angles are required to ensure a proper geometry of the metal center. While we have performed all simulations with GAFF2, our parameters are conceptually compatible with the older GAFF, but they require additional testing. However, since the parameters for bonds and angles including the atoms for which we define our custom parameters (carbons in pure aromatic systems and sp^2^ nitrogens in pure aromatic systems) are comparable between GAFF and GAFF2, we expect our parameters to work well with both. A detailed description of all nonstandard parameters alongside explanations for each adjustment is listed in the Supporting Information, Section S1. All parameters are also listed in the rh1.frcmod and rh3.frcmod files provided as Supporting Information.

**Table 1:** Bond Parameters for the Rh^I^ and Rh^III^ Complexes. Force constants given in kcal/mol/Å^2^

	Rh^I^		Rh^III^	
bond	*k*_r_	*r*_0_/Å	*k*_r_	*r*_0_/Å
M1–Y1	58.6	2.16	66.6	2.08
M1–Y6	89.8	2.06	70.2	2.09
Y1–Y1	354.25	1.45	354.25	1.47
Y6–YC	386.49	1.36	386.49	1.34
YC–YC	354.25	1.40	354.25	1.47
M1–Y8	N/A	N/A	81.4	2.41

**Table 2:** Angle Parameters for Rh^I^ and Rh^III^ Complexes. Force constants given in kcal/mol/rad^2^

	Rh^I^		Rh^III^	
angle	*k*_θ_	θ_0_/deg	*k*_θ_	θ_0_/deg
Y1–M1–Y6	30.00	143.00	30.00	129.00
Y6–M1–Y6	139.49	108.00	154.06	84.00
M1–Y6–ca	194.59	125.16	139.36	123.71
M1–Y6–YC	194.59	126.00	139.36	118.00
Y1–M1–Y8	N/A	N/A	30.00	125.00
Y6–M1–Y8	N/A	N/A	30.00	101.00

### Molecular Dynamics Simulation Protocol

The MD simulations are performed with the PMEMD implementation of the SANDER simulation engine and its CUDA-variant^91–93^ included in AmberTools23/Amber22.^58,59^ The Rh complex is described with the GAFF2 force field and the additional parameters presented in Tables 1 and 2, and placed in a truncated octahedron of OPC^94^ water with a minimum distance between any solute atom and the box border of 20 Å. The resulting number of water molecules thus varies according to the complex size; there are 3295 water molecules in the simulations with Rh^I^-bpy, and there are 4368 in those with Rh^III^-dppz. The positively charged complexes are neutralized by the addition of one chloride ion. For all simulations, a time step of 2 fs is used. To enable such a long time step, the SHAKE algorithm^95^ is used to freeze the lengths of bonds containing hydrogen at a relative geometrical tolerance of 1 × 10^–7^. Constant pressure is applied using isotropic pressure scaling employing the Berendsen barostat. The cutoff for nonbonded interactions is set to 10 Å. Before simulation, the systems are minimized for a total of 10,000 steps, using a steepest descent algorithm for the first 5000 steps and a conjugate gradient algorithm for the second 5000 steps. During the minimization, the SHAKE algorithm is disabled. Following minimization, the systems are heated from 0 to 300 K in 100 ps (50,000 steps) and then equilibrated at 300 K for 10 ns (5,000,000 steps). The temperature is controlled using a Langevin thermostat at a collision frequency of 1 ps^–1^. Subsequently, a 100 ns (50,000,000 steps) trajectory is produced for each complex, and 10,000 time steps (one every 10 ps) are used for the analysis.

## Results and Discussion

This section is organized as follows. First, we validate the force field parameters for Rh^I^-bpy and Rh^III^-bpy by comparing characteristic bonds, angles, and dihedrals between structures from the MD simulations with the B3LYP reference equilibrium geometry, as well as by comparing energy profiles computed with the force field to energy profiles computed with B2PLYP. After establishing that the force field accurately represents the geometry of bpy-based Rh complexes (in the following abbreviated as Rh-bpy), we test its transferability to other α-diimine-based Rh complexes by comparing the geometries of Rh-bpy to the respective complexes containing phenanthroline (Rh-phen) and dipyrido­[3,2-*a*:2′,3′-*c*]­phenazine (Rh-dppz). Finally, we assess the applicability of the force field for investigating the interaction with the environment by analyzing the electrostatics of the complex, including their 3D solvation shells.

### Validation of the Rh-bpy Parameters

To assess how accurately the force field reproduces the geometries of Rh^I^-bpy and Rh^III^-bpy, their characteristic bond lengths, angles, and dihedrals are presented in Figure 3.

**Figure 3:**
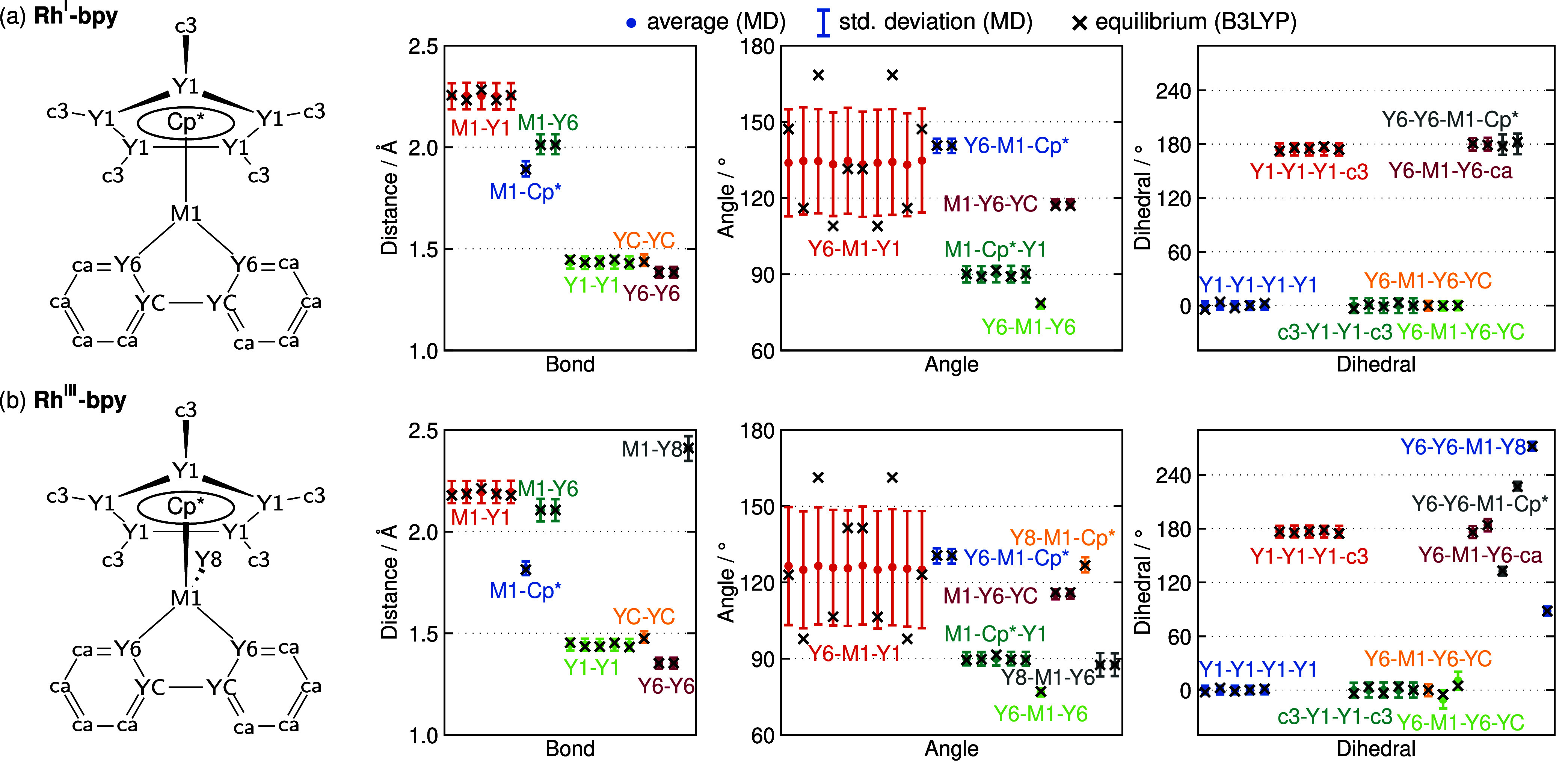
Comparison of characteristic bond lengths, angles, and dihedrals between the force field MD simulations (dots for averages, error bars for the standard deviation) and the B3LYP reference equilibrium values (black x’s) for (a) Rh^I^-bpy and (b) Rh^III^-bpy. The structures on the left side indicate the atom types used for labeling.

As one can see, the force fields accurately reproduce the equilibrium values of the B3LYP reference, with all total errors between the average and the reference below 0.01 Å or 1°, for distances or angles and dihedrals, respectively. As explained above, the average values do not necessarily correspond to the equilibrium parameters that define the force field due to the antagonistic effect of some parameters. For instance, the M1–Y1 bonds, which describe the bonding interactions between Rh and the aromatic carbons in Cp*, are parametrized at 2.16 Å for Rh^I^-bpy, but the force field accurately reproduces 2.25 Å of the B3LYP reference. Furthermore, the force field exhibits symmetry for the relevant parameters, including the M1–Y1 bonds, Y6–M1–Y1 or M1–Cp*–Y1 angles, and the three sets of dihedrals including Y1 atoms. Crucially, the parameters are even symmetric in the force field MD dynamics, when they are not symmetric in the frozen B3LYP reference equilibrium structure. The full distribution profiles of each parameter can be found in Section S2 of the Supporting Information.

One parameter that deserves further discussion from Figure 3 is the Y6–M1–Y1 angle, which describes the relative arrangement of the Cp* and bpy ligands across the metal center. The full distributions are shown in Figure 4. As there are 2 Y6-nitrogens and 5 Y1-carbons, there are a total of 10 distinct Y6–M1–Y1 angles in both oxidation states of the complex. Notably, the histograms of these 10 angles deviate from the symmetric bell-shape observed for most other parameters. Thus, the standard deviation shown in Figure 3 does not properly capture the range over which Y6–M1–Y1 angles occur, which necessitates analysis of the actual probability distributions. In the B3LYP reference geometry, the 10 angles correspond to 5 symmetry-unique pairs of symmetry equivalent angles. In the simulations, thermal motion breaks this static symmetry, leading to three distinct peaks in the angular distribution of Rh^I^-bpy, and four or five in the case of Rh^III^-bpy. Importantly, in both oxidation states, the 10 profiles are identical to each other, demonstrating the free rotation of the Cp* ligand and confirming that the η^5^ nature of the bond is correctly captured by the σ-bonded model.

**Figure 4:**
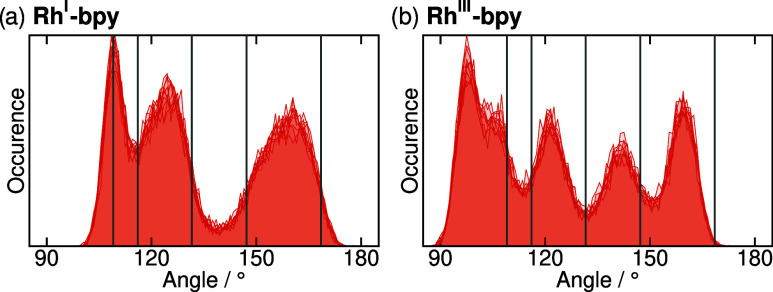
Distribution profiles of the Y6–M1–Y1 angle in the MD simulations of (a) Rh^I^-bpy and (b) Rh^III^-bpy. Each panel shows 10 distinct distributions. The vertical lines show the five symmetry-unique values of the B3LYP reference equilibrium structure.

Up to this point, average bond lengths, angles, and dihedrals were compared to the equilibrium values obtained from the B3LYP reference geometry. While this confirms that the force field reproduces the overall structure, it does not account for distortions from the equilibrium geometry that happen during dynamics. To evaluate these effects, we compare energy profiles along key modes obtained with the force field to those from the high-level double-hybrid B2PLYP functional. This comparison directly evaluates the force field’s ability to represent the energetic landscape of the complex. These profiles are shown in Figure 5 for Rh^I^-bpy.

**Figure 5:**
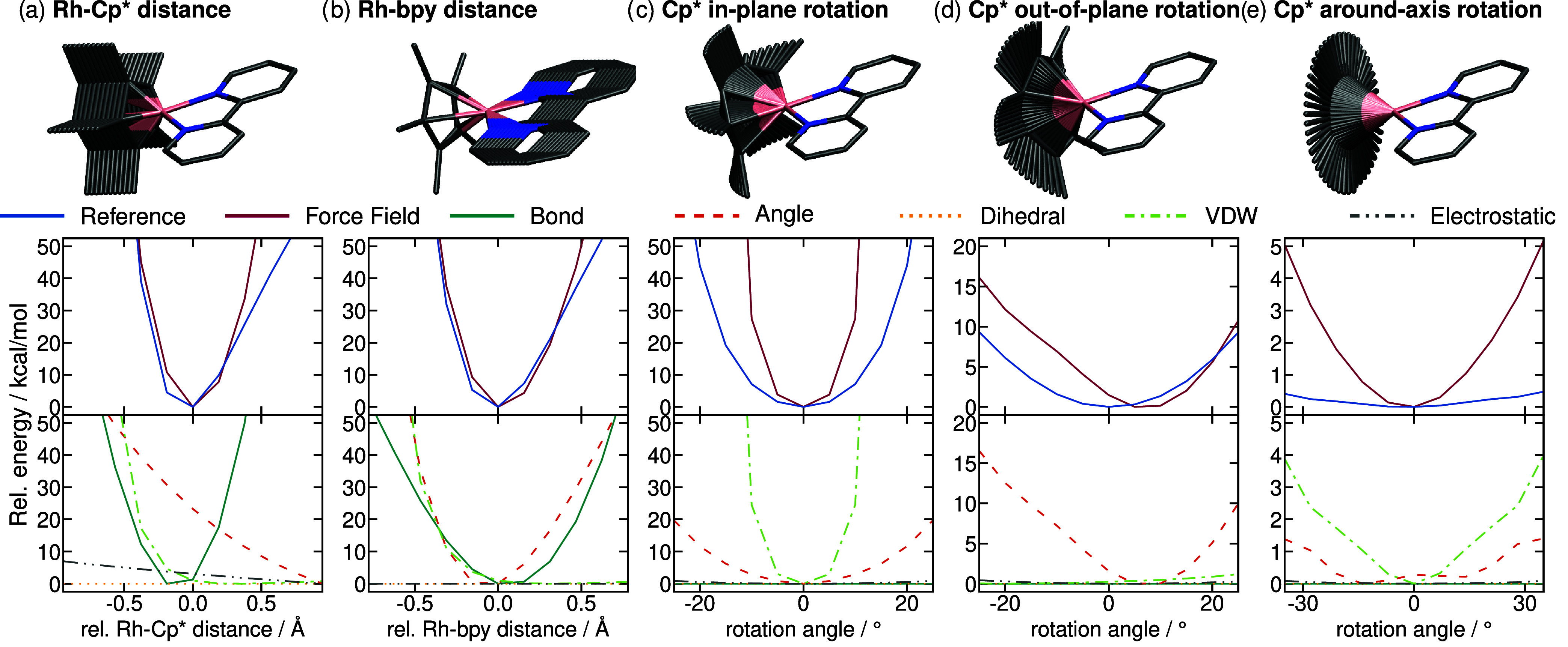
(a–e) Potential energy surfaces of Rh^I^-bpy calculated along key ligand displacement coordinates. The molecular structures highlight the scanned mode. The upper profiles compare energetic profiles obtained with the force field (red lines) against the B2PLYP reference (blue lines). The lower profiles show the individual contributions of the force field. Horizontal axes are shown relative to the equilibrium structure.

The B2PLYP distance profiles (Figure 5a,b) are reproduced very well by the force field. In both cases, the decomposition into force field terms shows that this good agreement is not due to an ideal equilibrium bond constant, but rather to the antagonistic effect that the equilibrium bond constant and angle constant have on each other. Especially the Rh–Cp* distance profile (Figure 5a) illustrates how the angle terms produce forces pushing the Cp* ligand away from the Rh center. To counteract these forces, the equilibrium bond constant is reduced compared to the equilibrium distance, which causes the overall force field minimum to coincide with the B2PLYP reference. While for Rh–Cp* the equilibrium bond constant is larger than the equilibrium distance, this effect is reversed for the Rh-bpy distance (Figure 5b), as here the angle terms are not dominated by the Cp*–Rh-bpy angle, but by the N–Rh–N angle, which biases the system toward smaller Rh-bpy distances. Additionally, van der Waals terms add a penalty to reduced distances, showing the interplay between many force field terms, which ultimately make up the final profile. These van der Waals terms are not parametrized for individual bonds, but have a more universal parametrization and thus were not adjusted in the force field parametrization.

In contrast to the distance profiles, the B2PLYP angle profiles are less accurately reproduced by the force field (Figure 5c–e), even though the overall trends match. This discrepancy comes mostly from van der Waals terms, which result in energy penalties along the Cp* in-plane (Figure 5c) and around-axis (Figure 5e) rotations. The B2PLYP scan of the around-axis rotation (Figure 5e) further shows the effectively barrierless rotation of Cp*, highlighting the need for symmetrical parameters for this ligand. At the same time, the angle terms for the Cp* in-plane (Figure 5c) and out-of-plane (Figure 5d) rotations are effectively both dominated by the Y1–M1–Y6 (C–Rh–N) angle, which means that the in-plane and out-of-plane rotations cannot be fine-tuned individually. Furthermore, the σ-bonded approach, where the η^5^ bond is represented by five individual Rh–C bonds, results in the out-of-plane rotational minimum being offset from the B2PLYP reference.

Due to the symmetry of the complex, the three rotations of the Cp* ligand in Rh^I^-bpy also cover the rotation of the bpy ligand. However, in the case of Rh^III^-bpy, due to the added chloride ligand, translating and rotating each of the 3 ligands results in 11 unique scans, which are presented in Figure 6. Somehow, the larger number of parameters resulting from the introduction of a third ligand gives increased control over the geometry of the complex. Thus, the B2PLYP energy surfaces are better reproduced by the force field for Rh^III^-bpy than for Rh^I^-bpy. The only profiles exhibiting significant differences between B2PLYP and the force fields are the in-plane rotations of Cp* and bpy as well as the Rh–Cl distance. In the case of the rotations (Figure 6b,f), the force field produces too high energies due to van der Waals interactions, similar to the Rh^I^-bpy case. In the case of the Rh–Cl bond (Figure 6i), increased distances come attached with an electrostatic penalty due to the increased charge separation. Still, as this contribution scales effectively linearly with the distance, the harmonic nature of the bond is preserved. In conclusion, these scans indicate that the force field reproduces the B2PLYP potential energy surfaces of both Rh^I^-bpy and Rh^III^-bpy satisfactorily.

**Figure 6:**
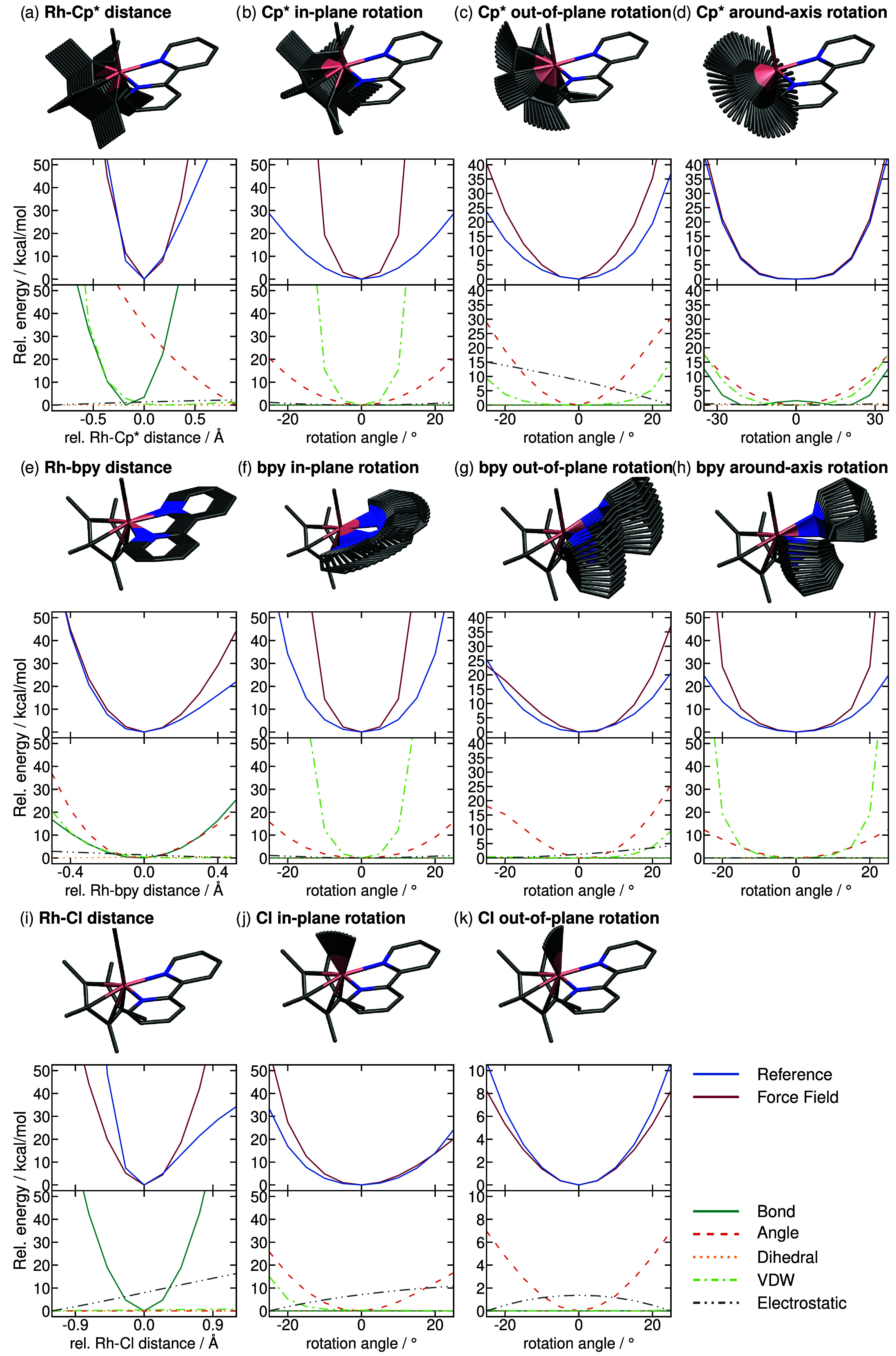
(a–k) Potential energy surfaces of Rh^III^-bpy calculated along key ligand displacement coordinates. The molecular structures highlight the scanned mode. The upper profiles compare energetic profiles obtained with the force field and the B2PLYP reference. The lower profiles show the individual contributions of the force field. Horizontal axes are shown relative to the equilibrium structure.

### Transferability

So far, the force field results have been compared to reference data for the Rh^I^-bpy and Rh^III^-bpy complexes, for which the force field parameters were specifically optimized. We now evaluate the transferability of these parameters to other 2,2′-bipyridyl (α-diimine) ligands, using phen and dppz as examples (see Section S3 for a guide on how to parametrize any complex of this class). Table 3 presents relevant bond lengths, angles, and one dihedral, showing the averages from the MD simulations and deviations from the B3LYP reference. The values for Rh-bpy are included for comparison.

**Table 3:** Averages (and Errors with respect to the Optimized B3LYP Geometry) for Selected Bond Lengths, Angles, and Dihedrals in the Two Oxidation States of Each of the Three Complexes^a^

	bpy		phen		dppz	
parameter	Rh^I^	Rh^III^	Rh^I^	Rh^III^	Rh^I^	Rh^III^
M1–Cp*	1.894 (0.003)	1.820 (0.007)	1.888 (0.003)	1.819 (0.012)	1.892 (0.011)	1.819 (0.012)
M1–Y1	2.251 (0.000)	2.195 (0.007)	2.247 (−0.001)	2.195 (0.010)	2.250 (0.006)	2.194 (0.010)
M1–Y6	2.015 (0.004)	2.106 (−0.001)	2.027 (0.001)	2.128 (0.005)	2.028 (0.006)	2.120 (−0.001)
Y1–Y1	1.433 (−0.006)	1.443 (0.002)	1.433 (−0.006)	1.444 (0.002)	1.433 (−0.006)	1.444 (0.002)
Y6–YC	1.385 (0.001)	1.353 (0.000)	1.373 (−0.010)	1.344 (−0.014)	1.376 (−0.005)	1.346 (−0.007)
YC–YC	1.444 (0.008)	1.482 (0.008)	1.420 (0.015)	1.459 (0.028)	1.421 (0.006)	1.458 (0.010)
M1–Y8	N/A	2.409 (−0.002)	N/A	2.405 (−0.006)	N/A	2.409 (−0.002)
Y6–M1–Cp*	140.5 (−0.1)	130.4 (−0.3)	140.8 (0.6)	130.6 (0.1)	140.8 (0.5)	130.4 (−0.1)
Y6–M1–Y6	77.8 (−0.8)	76.7 (−0.4)	77.4 (−2.2)	76.6 (−1.4)	77.3 (−2.0)	76.5 (−1.3)
M1–Y6–YC	117.75 (0.8)	115.4 (−0.7)	117.2 (2.5)	114.9 (1.1)	117.3 (2.0)	115.1 (0.8)
Y8–M1–Cp*	N/A	127.0 (0.2)	N/A	127.3 (0.4)	N/A	127.1 (1.2)
Y8–M1–Y6	N/A	87.7 (0.1)	N/A	87.1 (−0.2)	N/A	87.5 (0.3)
Y1–Y1–Y1–c3	174.3 (−0.7)	176.7 (0.2)	175.0 (−0.1)	177.0 (0.4)	174.9 (−0.3)	176.9 (0.2)

a Distances in Å, angles and dihedrals in degrees.

Because Rh-phen and Rh-dppz employ the same parameters as Rh-bpy but differ in their reference geometries, larger deviations are expected. Nevertheless, bond distances are well reproduced across all complexes, with errors below 0.015 Å, while bond angles show slightly larger deviations. For Rh-phen and Rh-dppz, the N–Rh–N and Rh–N–C angles deviate by up to 3°, slightly distorting the 5-membered Rh–N ring, but the overall geometry is well reproduced. Because most dihedral angles are centered around either 0 or 180°, a comparison of their mean values is not informative. Accordingly, Table 3 includes only the offset of the Cp*-methyls from the Cp*-ring plane (Y1–Y1–Y1–c3). In free Cp* molecules, the averages of this dihedral axis should be 180°. However, due to nonbonded interactions in the complex, the methyl groups are pulled toward the metal center, reducing the dihedral. Although this feature is mostly governed by nonbonded interactions, which are not explicitly parametrized here, it is captured with remarkable accuracy, with errors below 1°.

Another interesting dihedral is the 3–2–2′–3′ (ca–YC–YC–ca) dihedral of the pyridine-based ligands, which describes how well the two pyridine rings align within the same plane. This dihedral distribution is indicative of the structural flexibility of the pyridine-based ligand, and thus, the overall dihedral distribution is much more informative than the mere average, which is why the full distribution is shown for all three complexes in Figure 7. It is a known characteristic of bipyridine to be more flexible due to the repulsion of the two hydrogens facing each other in the 3 and 3′ positions, compared to the ligands where the atoms bonded to the 3 and 3′ positions are in turn bonded to each other, as it happens in phen and dppz. While the dihedral profiles at both oxidation states are very similar for Rh-phen and Rh-dppz, as expected, there is a clear distinction to Rh-bpy, which shows a much broader profile. Especially for Rh^III^-bpy, the occurrence of perfectly planar geometries is about 25% less frequent than for the other two compounds, and the distribution exceeds ±20° at both oxidation states, which is also not the case for Rh-phen or Rh-dppz.

**Figure 7:**
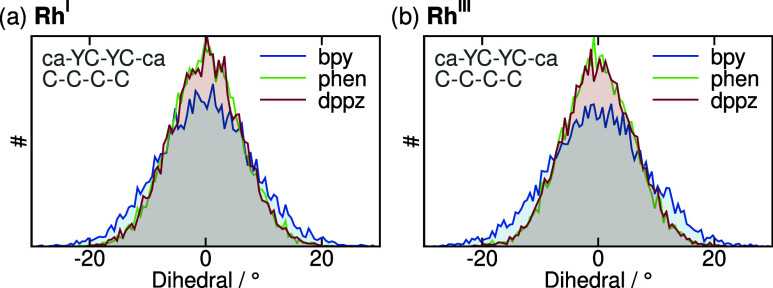
Dihedral between the two pyridine rings in the three different α-diimine ligands for (a) Rh^I^ and (b) Rh^III^.

Overall, we can conclude that the force field parameters describe the geometries of the three different complexes with good accuracy, underscoring their transferability across different ligands.

### Application

We next illustrate the force field’s applicability to solvation, taking water as a representative case. In an Amber-style force field, nonbonded interactions are defined either by van der Waals (Lennard-Jones) terms (using standardized parameters from GAFF2)—or by Coulomb interactions, governed by the partial charges of each individual atoms. Regardless of the chosen force field and its parameters, it is essential (or at least strongly recommended) to compute these partial charges for every nonstandard molecule included in a simulation. In this work, restricted electrostatic potential (RESP) charges are used, as these are designed to reproduce the electrostatic potential of a molecule, but alternative charge methods might be preferable in other contexts. Technically, the charges are not a measure of the force field’s quality, as they are derived from quantum chemical calculations. Still, to estimate the effect of the chosen charge method, we compared our RESP charges with charges from a natural population analysis (NPA) in the Supporting Information, Section S4. The results show that the two methods, while producing significantly different charges, barely affect the geometry of the complex, thus validating our force field parameters for use with different charge methods. Nevertheless, we encourage the use of appropriate charge methods in different contexts.

Figure 8 shows the RESP charges parametrized for the two oxidation states of the three complexes. The overall charge difference between the neutral Rh^I^ complex and the positively charged Rh^III^ complex is only 1, because the latter complex contains one additional negative charge introduced by the chloride. The most notable difference between the two oxidation states is the charge of the Rh center, which is close to −0.7 atomic charge units in Rh^I^, and just about −0.3 atomic charge units in Rh^III^ in all three different complexes. However, this increase in positive charge is almost completely compensated by the −0.4 charge on the chloride ligand, implying that the remaining positive charge must be delocalized over the Cp* and bpy/phen/dppz ligands. Approximately half of this charge (+0.49 in the case of Rh^III^-bpy, with comparable values in the other two complexes) is distributed over the Cp* ligand. The charge increase is mostly localized on the 5 aromatic carbons and the 15 hydrogens, while the 5 methyl carbons become slightly more negative. This redistribution increases the polarization of the bonds in the Cp* ligand. Another +0.42 charge increase is distributed over the bpy ligand, and this increase is similar for phen and dppz. However, due to the different atomic scaffolds, the charge distribution between bpy, phen, and dppz is quite different even on the atoms shared between these three ligands. In summary, the first positive charge introduced by oxidizing Rh is compensated by the added negative charge of the chloride, while the second positive charge is delocalized in roughly equal parts on the Cp* and the bpy/phen/dppz ligands. In both oxidation states, the metal center carries a negative partial charge and the dppz ligand carries a high degree of polarization on the two rings not directly next to the metal center.

**Figure 8:**
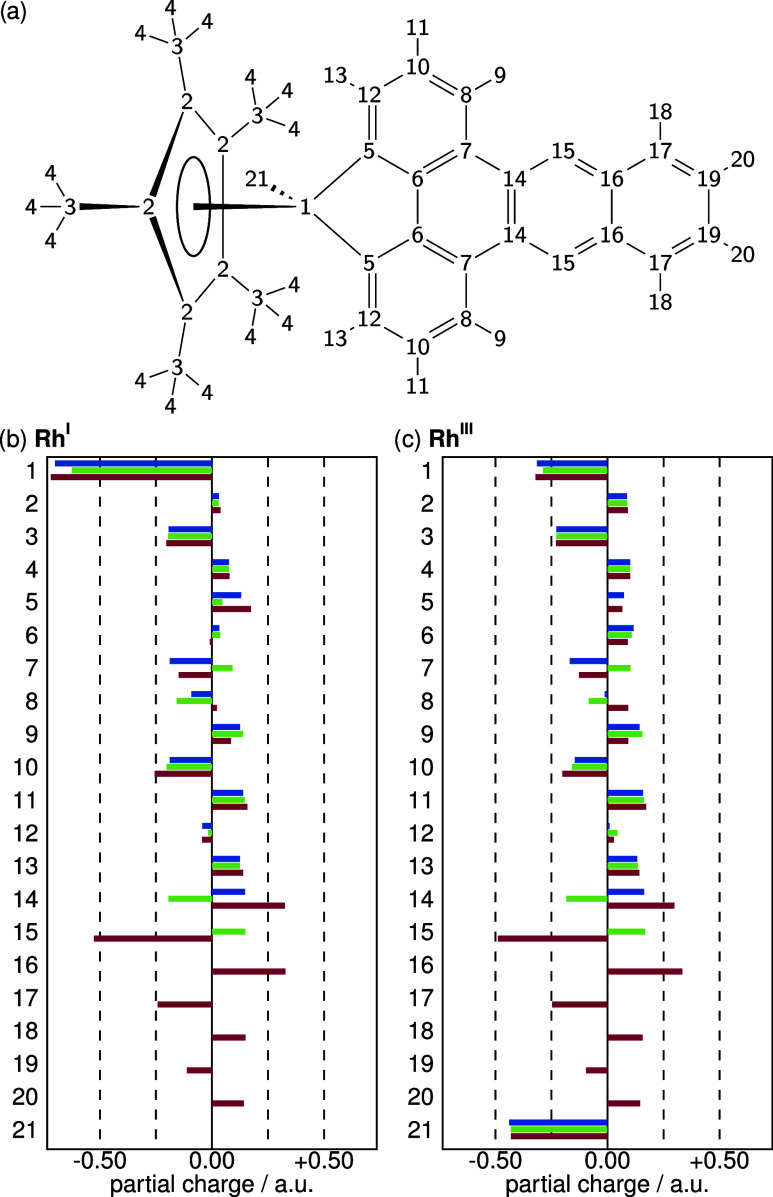
(a) Numbering scheme for the atoms in the Rh complexes. (b, c) RESP charges in atomic units for the Rh-bpy (blue), Rh-phen (green), and Rh-dppz (red) complexes in the Rh^I^ (b) and Rh^III^ (c) oxidation states.

Naturally, these different charge distributions influence the interactions between the complex and its environment. Figure 9 illustrates two complementary representations of the solvent distribution around the complex. On the left side of each panel, the one-dimensional radial distribution function (1D-RDF) of hydrogens and oxygens around the Rh center is shown, providing a measure of the relative density of the respective species in spherical bins around the metal center. The right side displays two views of the three-dimensional spatial distribution function (3D-SDF), which maps the average number of atoms within specific volume elements per frame. Our volume elements are cubes of 0.2 Å side length, such that on average, roughly 0.00027 water molecules should be in each volume element at each individual frame. This average can be computed from the volume element and the molar density of water.^96^ Shown in Figure 9 are all regions where the water density is at least 5 times higher than the average, i.e., where the atoms comprising water molecules are more than 5 times more likely to be than in the bulk (0.00135 atoms per cell for oxygen, 0.00270 for hydrogen atoms).

**Figure 9:**
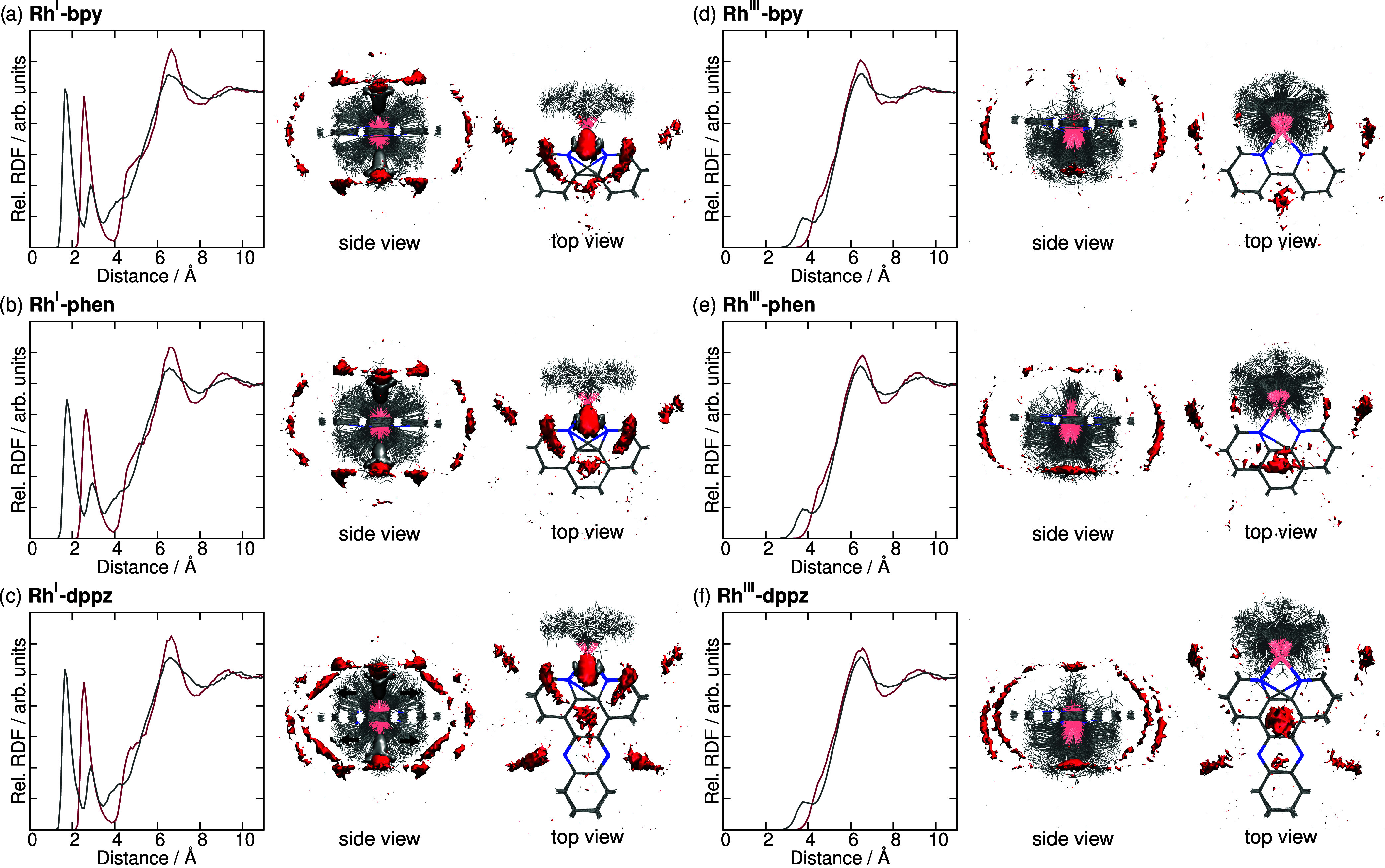
(a–f) One-dimensional (1D) radial distribution functions (left) around the Rh center and 3D spatial distribution functions (right) of hydrogens (gray) and oxygen (red) atoms for the three investigated complexes at the two different oxidation states. For the 3D distributions, visual cutoffs are set to 5 times the expectation value. In the side view of Rh^I^-dppz, black arrows indicate highly localized hydrogen distributions.

Starting with Rh^I^-bpy (Figure 9a), the hydrogen RDF quickly rises sharply beyond 1 Å and reaches a maximum indicating the first solvation shell with a tight hydrogen bond to the metal center. This species can be considered a precursor to the Rh–H^26,27^ species that is formed by proton abstraction from water. About 1 Å further, the oxygen RDF shows a peak, followed by a smaller hydrogen peak, which arises from the oxygen and the second hydrogen atom of the water molecule that forms the first hydrogen peak. In the 3D-SDF, this arrangement is also evident, with the first hydrogen at closer distances and oxygen at larger distances from the Rh. At distances beyond 5 Å from the metal center, the RDFs for both hydrogen and oxygen rise again, peaking just above 6 Å, before converging to the bulk density at around 10 Å. This second peak corresponds to a second solvation shell, which appears in the 3D-SDFs as a half moon above and below the bpy ligand. This shell partially overlaps in distance with another solvation region, corresponding to oxygen atoms positioned on the sides of the complex not occupied by either the ligands or the first solvation shell. This third solvation shell is not hydrogen binding, as it does not clearly include hydrogen. Rather, it could be oxygen binding to the positively polarized hydrogens of Cp* and bpy. Similar O-binding to the positively polarized H atoms of the Cp* ring has been computationally found for the carbamoyl O atom of nicotineamides.^97^

The solvent distributions around Rh^I^-phen and Rh^I^-dppz (Figure 9b,c) are very similar to that of Rh^I^-bpy, and since the electrostatics of all three complexes are very similar at least around the metal center, the RDFs are almost identical. However, the 3D-SDFs of Rh^I^-dppz reveal additional areas of high oxygen density next to the additional nitrogens of the dppz ligand, which orient in a roughly 45° angle above and below the molecular plane. This bonding is actually achieved by hydrogen bonds, and the respective hydrogen atoms are highly constrained, in short proximity (ca. 2 Å) to the nitrogen atoms. As a result of this narrow confinement, the respective density blobs in Figure 9c are barely visible, which is why they are marked with black arrows. This strong hydrogen bonding is due to the strong backbonding of the Rh^I^ center into the phenanzine-N-dominated lowest unoccupied molecular orbital (LUMO) of the dppz ligand, giving rise to the elevated negative charge on these N atoms (recall atom 15 in Figure 8b) and thus making them prone to hydrogen bonding with water.^98^

Upon oxidation (Figure 9d–f), the water molecules that directly hydrogen-bond to the metal center disappear, as do the peaks at short distances in the RDF. This is due to the reduced negative charge at the Rh^III^ center (see Figure 8). However, the areas of increased oxygen density close to the hydrogens in Cp* confirm that this binding occurs to the positively polarized hydrogens (atom number 4 in Figure 8a, charged between +0.07 and +0.10 in the different systems).^97^ A second density region remains near the 3 and 3′ hydrogens, but is primarily localized under the bpy plane—the side toward which the Cp* is tilted, again suggesting binding interactions with these hydrogens. This second region is even more pronounced in Rh^III^-phen than in Rh^III^-bpy, located directly below the added third ring. These densities are also present for Rh^III^-dppz, and additionally, the areas of elevated oxygen concentrations arising from hydrogen bonding to the nitrogens are preserved. However, this oxygen binding is less prominent in Rh^III^-dppz compared to Rh^I^-dppz, as the backbonding into the LUMO of the dppz ligand is weakened upon oxidation, which slightly diminishes the partial charge on the nitrogens from −0.53 to −0.49 (recall atom 15 in Figure 8c). Albeit only a small difference, this reduction in charge leads to a 67% reduction of hydrogen concentration at distances below 2.5 Å from the nitrogens. As a consequence of the weakened hydrogen bonding, the areas of elevated oxygen density are also less structured, and the split into four 45° regions above and below the molecular plane is not retained. There are additional regions of increased water density beyond those shown here as the cutoffs highlight only areas where the water density is at least 5 times the bulk value. This should not be taken to mean that water does not interact with the oxidized complex. Nevertheless, it is clear that Rh^I^ attracts and binds water much more effectively than Rh^III^.

## Conclusions

We report force field parameters for pentamethylcyclopentadienyl Rh^I^ and Rh^III^ complexes with α-diimine ligands, fully compatible with the general Amber force field and transferable to related complexes of the same family. The parametrized force field reliably reproduces both the geometry and the intramolecular energetic landscape of these complexes, as evidenced by comparisons to a quantum chemical reference. Beyond static properties, we demonstrate the utility of the newly developed force field in capturing dynamic interactions with the environment, as exemplified by water. This work provides a versatile computational framework for exploring the structure, dynamics, and solvation of such complexes, providing insights relevant to catalysis in complex environments. By providing accurate parameters, which are transferable to Rh complexes with different α-diimine ligands, this study paves the way for predictive modeling of Rh-based catalysts and functional materials under realistic conditions.

## Supplementary Material





## Data Availability

Force field modification files for the [(bpy)­Rh­(Cp*)­Cl]^+^ and [(bpy)­Rh­(Cp*)] complexes are available for download as Supporting Information.

## Notes

Details on the parameters as well as a parametrization guide (PDF)
Force field modification (frcmod) files (ZIP)
