# Supplementary material for: Tailored GAFF Parameters for Pentamethylcyclopentadienyl Rh(I/III) Complexes with α‑Diimine Ligands: Validation and Solvation Studies
Source: J Phys Chem B. 2025 Dec 23;130(1):472–85. doi: 10.1021/acs.jpcb.5c07040 (PMC12794178; doi:10.1021/acs.jpcb.5c07040)
Supplement: Supplementary file 1 [file jp5c07040_si_001.pdf]

Supporting Information to: Tailored GAFF Parameters for Pentamethylcyclopentadienyl Rh(I/III) Complexes with α-diimine ligands: Validation and Solvation Studies

Richard Jacobi[1,2], Konstantinos P. Zois[1,2], Alexander K. Mengele[3], Sven Rau[3] and Leticia González[2,4]

December 2025

[1] Doctoral School in Chemistry (DoSChem), University of Vienna
Währinger Straße 42, 1090 Vienna, Austria

[2] Institute of Theoretical Chemistry, Faculty of Chemistry, University of Vienna
Währinger Straße 17, 1090 Vienna, Austria

[3] Institute of Inorganic Chemistry I, Ulm University
Albert-Einstein-Allee 11, 89081 Ulm, Germany

[4] Vienna Research Platform on Accelerating Photoreaction Discovery, University of Vienna
Währinger Straße 17, 1090 Vienna, Austria

Contents

S1 Non-standard parameters **S3**

S1.1 Custom atom types S3

S1.2 Rh(I) S3

S1.2.1 Bonded parameters S3

S1.2.2 Angle parameters S3

S1.2.3 Dihedral parameters S4

S1.2.4 Improper dihedrals S6

S1.3 Rh(III) S6

S1.3.1 Bonded parameters S6

S1.3.2 Angle parameters S6

S1.3.3 Dihedral parameters S7

S1.3.4 Improper dihedrals S9

S2 Full histograms of bond lengths, angles and dihedrals **S10**

S3 Parametrization guide **S13**

S4 Comparison of RESP and NPA charges **S14**

S1 Non-standard parameters

S1.1 Custom atom types

Except for the M1 atom type, which describes the Rh center, all custom atom types correspond to atom types in GAFF/GAFF2, and the parameters for mass and non-bonded interactions are directly taken from the corresponding GAFF atom type. Here is an overview over all atom types and the corresponding GAFF2 types:

Custom atom type	GAFF2 type	Element symbol
Y1	ca	C
Y6	nb	N
Y8	cl	Cl
YC	ca	C

For M1, mass and non-bonded parameters were assigned by MCPB for Rh. The mass is 102.91 atomic mass units, the van der Waals radius is 1.4645 Å, and the 6-12 potential well depth is 0.053 kcal/mol.

S1.2 Rh(I)

S1.2.1 Bonded parameters

In Amber style force fields, bonds are represented as:

$$E_{\text{bond}} = \sum_{\text{bonds}} k_b (r - r_0)^2. \tag{1}$$

Bond	k_b / kcal/mol/Å^2	r_0 / Å	Explanation
M1-Y1	58.6	2.16	1.
M1-Y6	89.8	2.06	1.
Y1-Y1	354.25	1.45	2.
Y1-c3	243.91	1.5147	3.
Y6-YC	386.49	1.36	2.
Y6-ca	386.49	1.3392	3.
YC-ca	354.25	1.3968	3.
YC-YC	354.25	1.40	2.

1. k_b is symmetrized (averaged) from MCPB parametrization, r_0 is defined manually.
2. k_b is taken from GAFF2 for the respective atom types, r_0 is defined manually.
3. Both k_b and r_0 are taken from GAFF2 for the respective atom types.

S1.2.2 Angle parameters

In Amber style force fields, angles are represented as:

$$E_{\text{angles}} = \sum_{\text{angles}} k_\theta (\theta - \theta_0)^2. \tag{2}$$

Angle	k_θ / kcal/mol/rad^2	θ_0 / °	Explanation
M1-Y1-Y1	0 00	0.00	1.
M1-Y1-c3	0.00	0.00	1.
M1-Y6-ca	194.59	125.16	2.
M1-Y6-YC	194.59	126.00	3.
Y1-M1-Y1	0.00	0.00	1.
Y1-M1-Y6	30.00	143.00	4.
Y6-M1-Y6	139.49	108.00	5.
Y1-Y1-Y1	63.670	120.020	6.
Y1-Y1-c3	60.740	120.830	6.
Y1-c3-hc	43.610	110.630	6.
Y6-ca-ca	68.170	122.940	6.
Y6-YC-ca	68.170	122.940	6.
Y6-YC-YC	68.170	122.940	6.
Y6-ca-h4	50.100	116.020	6.
ca-Y6-ca	83.150	117.240	6.
YC-Y6-ca	83.150	117.240	6.
YC-ca-ca	63.670	120.020	6.
YC-YC-ca	63.670	120.020	6.
YC-ca-ha	44.90	119.88	6.

1. The Rh-C-C angles are implicitly by the Rh-C bonds, as these are are all assigned the same bonded parameters. Defining angles here would lead to an over-parametrization, thus all parameters are set to 0.

2. k_θ is symmetrized (averaged) from MCPB parametrization, θ_0 is taken from the optimized reference geometry.

3. k_θ is symmetrized (averaged) from MCPB parametrization, θ_0 is defined manually.

4. Both k_θ and θ_0 are defined manually to achieve the best agreement of the energy scans in addition to tuning the equilibrium values.

5. k_θ is taken directly from MCPB parametrization, θ_0 is defined manually.

6. Both k_θ and θ_0 are taken from GAFF2 for the respective atom types.

S1.2.3 Dihedral parameters

In Amber style force fields, dihedrals are represented as:

$$E_{\text{dihedrals}} = \sum_{\text{dihedrals}} V_n[1 + cos(n\phi - \gamma)], \tag{3}$$

where usually $V_n = h_n/m_n$.

Dihedral	m_n / -	h_n / kcal/mol	γ / °	ϕ / -	Explanation
M1-Y1-Y1-Y1	3	0.00	0.00	3.0	1.
M1-Y1-Y1-c3	3	0.00	0.00	3.0	1.
M1-Y1-c3-hc	3	0.00	0.00	3.0	1.
M1-Y6-ca-ca	3	0.00	0.00	3.0	1.
M1-Y6-YC-ca	3	0.00	0.00	3.0	1.
M1-Y6-YC-YC	3	0.00	0.00	3.0	1.
M1-Y6-ca-h4	3	0.00	0.00	3.0	1.
Y1-M1-Y1-Y1	3	0.00	0.00	3.0	1.
Y1-M1-Y1-c3	3	0.00	0.00	3.0	1.
Y1-M1-Y6-ca	3	0.00	0.00	3.0	1.
Y1-M1-Y6-YC	3	0.00	0.00	3.0	1.
Y1-Y1-Y1-Y1	4	14.5	180.0	2.0	2.
Y1-Y1-Y1-c3	4	14.5	180.0	2.0	2.
Y1-Y1-c3-hc	1	0.0	0.0	1.0	2.
c3-Y1-Y1-c3	4	14.5	180.0	2.0	2.
Y6-M1-Y1-Y1	3	0.00	0.00	3.0	1.
Y6-M1-Y1-c3	3	0.00	0.00	3.0	1.
Y6-M1-Y6-ca	3	0.00	0.00	3.0	1.
Y6-M1-Y6-YC	3	0.00	0.00	3.0	1.
Y6-YC-YC-Y6	4	14.5	180.0	2.0	2.
Y6-ca-ca-ca	4	14.5	180.0	2.0	2.
Y6-YC-ca-ca	4	14.5	180.0	2.0	2.
Y6-YC-YC-ca	4	14.5	180.0	2.0	2.
Y6-ca-ca-ha	4	14.5	180.0	2.0	2.
Y6-YC-ca-ha	4	14.5	180.0	2.0	2.
ca-Y6-ca-ca	2	9.6	180.0	2.0	2.
YC-Y6-ca-ca	2	9.6	180.0	2.0	2.
ca-Y6-YC-ca	2	9.6	180.0	2.0	2.
ca-Y6-YC-YC	2	9.6	180.0	2.0	2.
ca-Y6-ca-h4	2	9.6	180.0	2.0	2.
YC-Y6-ca-h4	2	9.6	180.0	2.0	2.
YC-ca-ca-ca	4	14.5	180.0	2.0	2.
ca-YC-ca-ca	4	14.5	180.0	2.0	2.
YC-YC-ca-ca	4	14.5	180.0	2.0	2.
ca-YC-YC-ca	4	14.5	180.0	2.0	2.
YC-YC-ca-ha	4	14.5	180.0	2.0	2.
YC-ca-ca-ha	4	14.5	180.0	2.0	2.

1. These parameters were set to 0 by MCPB (except those which need to be non-zero due to division, which are assigned random values). The respective dihedrals are already implicitly defined by bonds, angles and the connectivity.

2. All parameters are taken from GAFF2 for the respective atom types.

S1.2.4 Improper dihedrals

In Amber style force fields, impropers are represented as:

$$E_{\text{improper}} = \sum_{\text{dihedrals}} V_n[1 + cos(n\phi - \gamma)]. \tag{4}$$

Dihedral	V_n / kcal/mol	γ / °	ϕ / -	Explanation
Y1-Y1-Y1-c3	1.1	180.0	2.0	1.
Y6-ca-ca-ca	1.1	180.0	2.0	1.
Y6-YC-ca-ca	1.1	180.0	2.0	1.
YC-ca-ca-ha	1.1	180.0	2.0	1.

1. All parameters are taken from GAFF2 for the respective atom types, and were initially assigned by Antechamber.

S1.3 Rh(III)

S1.3.1 Bonded parameters

In Amber style force fields, bonds are represented as:

$$E_{\text{bond}} = \sum_{\text{bonds}} k_b(r - r_0)^2. \tag{5}$$

Bond	k_b / kcal/mol/Å^2	r_0 / Å	Explanation
M1-Y1	66.6	2.08	1.
M1-Y6	70.2	2.09	1.
M1-Y8	81.4	2.4110	2.
Y1-Y1	354.25	1.47	3.
Y1-c3	243.91	1.5147	4.
Y6-YC	386.49	1.34	3.
Y6-ca	386.49	1.3392	4.
YC-ca	354.25	1.3968	4.
YC-YC	354.25	1.47	3.

1. k_b is symmetrized (averaged) from MCPB parametrization, r_0 is defined manually.
2. Both k_b and r_0 are created using MCPB with the Seminario method.
3. k_b is taken from GAFF2 for the respective atom types, r_0 is defined manually.
4. Both k_b and r_0 are taken from GAFF2 for the respective atom types.

S1.3.2 Angle parameters

In Amber style force fields, angles are represented as:

$$E_{\text{angles}} = \sum_{\text{angles}} k_\theta(\theta - \theta_0)^2. \tag{6}$$

Angle	k_θ / kcal/mol/rad^2	θ_0 / °	Explanation
M1-Y1-Y1	0.00	0.00	1.
M1-Y1-c3	0.00	0.00	1.
M1-Y6-ca	139.36	123.71	2.
M1-Y6-YC	139.36	118.00	3.
Y1-M1-Y1	0.00	0.00	1.
Y1-M1-Y6	30.00	129.00	4.
Y6-M1-Y6	154.06	84.00	5.
Y1-M1-Y8	30.00	125.00	4.
Y6-M1-Y8	30.00	101.00	4.
Y1-Y1-Y1	63.670	120.020	6.
Y1-Y1-c3	60.740	120.830	6.
Y1-c3-hc	43.610	110.630	6.
Y6-ca-ca	68.170	122.940	6.
Y6-YC-ca	68.170	122.940	6.
Y6-YC-YC	68.170	122.940	6.
Y6-ca-h4	50.100	116.020	6.
ca-Y6-ca	83.150	117.240	6.
YC-Y6-ca	83.150	117.240	6.
YC-ca-ca	63.670	120.020	6.
YC-YC-ca	63.670	120.020	6.
YC-ca-ha	44.90	119.88	6.

1. The Rh-C-C angles are implicitly by the Rh-C bonds, as these are are all assigned the same bonded parameters. Defining angles here would lead to an overparametrization, thus all parameters are set to 0.

2. k_θ is symmetrized (averaged) from MCPB parametrization, θ_0 is taken from the optimized reference geometry.

3. k_θ is symmetrized (averaged) from MCPB parametrization, θ_0 is defined manually.

4. Both k_θ and θ_0 are defined manually to achieve the best agreement of the energy scans in addition to tuning the equilibrium values.

5. k_θ is taken directly from MCPB parametrization, θ_0 is defined manually.

6. Both k_θ and θ_0 are taken from GAFF2 for the respective atom types.

S1.3.3 Dihedral parameters

In Amber style force fields, dihedrals are represented as:

$$E_{\text{dihedrals}} = \sum_{\text{dihedrals}} V_n[1 + cos(n\phi - \gamma)], \tag{7}$$

where usually $V_n = h_n/m_n$.

Dihedral	m_n / -	h_n / kcal/mol	γ / °	ϕ / -	Explanation
M1-Y1-Y1-Y1	3	0.00	0.00	3.0	1.
M1-Y1-Y1-c3	3	0.00	0.00	3.0	1.
M1-Y1-c3-hc	3	0.00	0.00	3.0	1.
M1-Y6-ca-ca	3	0.00	0.00	3.0	1.
M1-Y6-YC-ca	3	0.00	0.00	3.0	1.
M1-Y6-YC-YC	3	0.00	0.00	3.0	1.
M1-Y6-ca-h4	3	0.00	0.00	3.0	1.
Y1-M1-Y1-Y1	3	0.00	0.00	3.0	1.
Y1-M1-Y1-c3	3	0.00	0.00	3.0	1.
Y1-M1-Y6-ca	3	0.00	0.00	3.0	1.
Y1-M1-Y6-YC	3	0.00	0.00	3.0	1.
Y1-Y1-Y1-Y1	4	14.5	180.0	2.0	2.
Y1-Y1-Y1-c3	4	14.5	180.0	2.0	2.
Y1-Y1-c3-hc	1	0.0	0.0	1.0	2.
Y6-M1-Y1-Y1	3	0.00	0.00	3.0	1.
Y6-M1-Y1-c3	3	0.00	0.00	3.0	1.
Y6-M1-Y6-ca	3	0.00	0.00	3.0	1.
Y6-M1-Y6-YC	3	0.00	0.00	3.0	1.
Y6-YC-YC-Y6	4	14.5	180.0	2.0	2.
Y6-ca-ca-ca	4	14.5	180.0	2.0	2.
Y6-YC-ca-ca	4	14.5	180.0	2.0	2.
Y6-YC-YC-ca	4	14.5	180.0	2.0	2.
Y6-ca-ca-ha	4	14.5	180.0	2.0	2.
Y6-YC-ca-ha	4	14.5	180.0	2.0	2.
Y8-M1-Y1-Y1	3	0.00	0.00	3.0	1.
Y8-M1-Y1-c3	3	0.00	0.00	3.0	1.
Y8-M1-Y6-ca	3	0.00	0.00	3.0	1.
Y8-M1-Y6-YC	3	0.00	0.00	3.0	1.
c3-Y1-Y1-c3	4	14.5	180.0	2.0	2.
ca-Y6-ca-ca	2	9.6	180.0	2.0	2.
YC-Y6-ca-ca	2	9.6	180.0	2.0	2.
ca-Y6-YC-ca	2	9.6	180.0	2.0	2.
ca-Y6-YC-YC	2	9.6	180.0	2.0	2.
ca-Y6-ca-h4	2	9.6	180.0	2.0	2.
YC-Y6-ca-h4	2	9.6	180.0	2.0	2.
YC-ca-ca-ca	4	14.5	180.0	2.0	2.
ca-YC-ca-ca	4	14.5	180.0	2.0	2.
YC-YC-ca-ca	4	14.5	180.0	2.0	2.
ca-YC-YC-ca	4	14.5	180.0	2.0	2.
YC-YC-ca-ha	4	14.5	180.0	2.0	2.
YC-ca-ca-ha	4	14.5	180.0	2.0	2.

1. These parameters were set to 0 by MCPB (except those which need to be non-zero due to division, which are assigned random values). The respective dihedrals are already implicitly defined by bonds, angles and the connectivity.

2. All parameters are taken from GAFF2 for the respective atom types.

S1.3.4 Improper dihedrals

In Amber style force fields, impropers are represented as:

$$E_{\text{improper}} = \sum_{\text{dihedrals}} V_n[1 + cos(n\phi - \gamma)]. \tag{8}$$

Dihedral	V_n / kcal/mol	γ / °	ϕ / -	Explanation
Y1-Y1-Y1-c3	1.1	180.0	2.0	1.
Y6-ca-ca-ca	1.1	180.0	2.0	1.
Y6-YC-ca-ca	1.1	180.0	2.0	1.
YC-ca-ca-ha	1.1	180.0	2.0	1.

1. All parameters are taken from GAFF2 for the respective atom types, and were initially assigned by Antechamber.

S2 Full histograms of bond lengths, angles and dihedrals

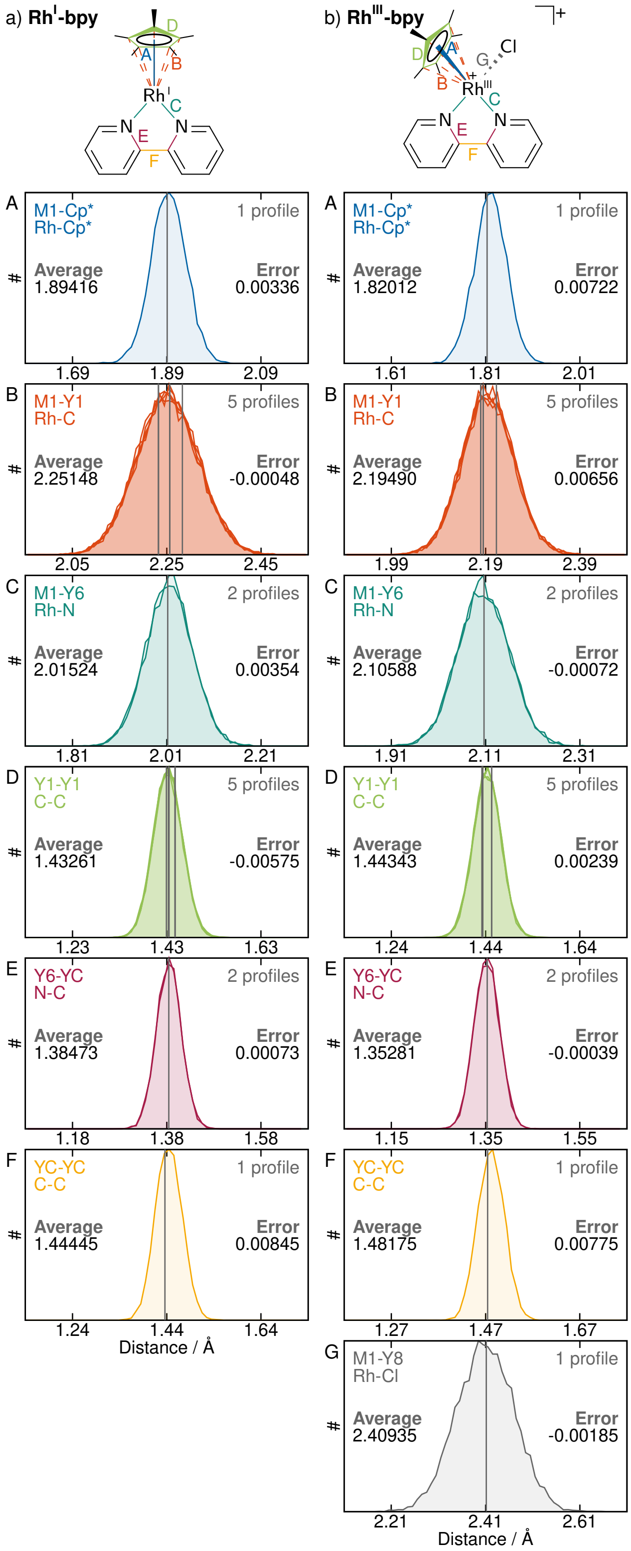

Figure S1: Characteristic bond lengths in a) Rh^{I}-bpy and b) Rh^{III}-bpy. The B3LYP reference values are shown as vertical lines. The bond definitions are given in terms of atom types according to Figure 2b (above) and element names (below). The number of profiles refers to how many different bonds of the same type are shown.

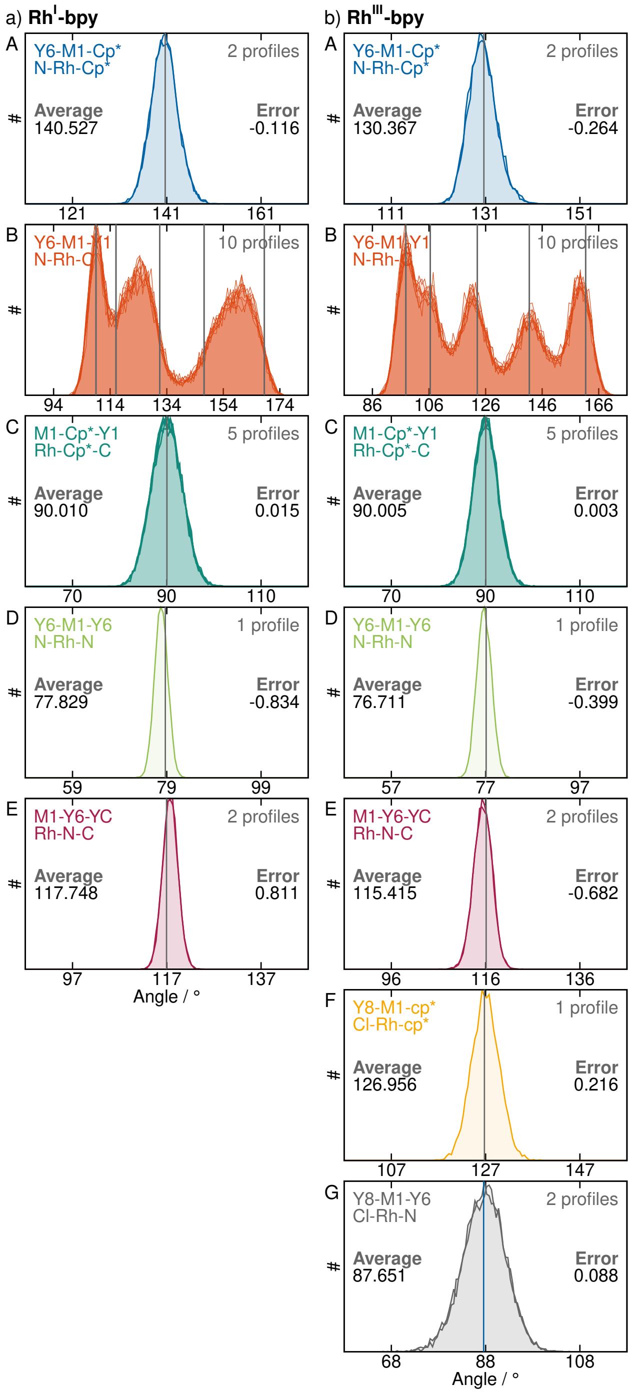

Figure S2: Characteristic angles in a) Rh^{I}-bpy and b) Rh^{III}-bpy. The B3LYP reference values are shown as vertical lines. The bond definitions are given in terms of atom types according to Figure 2b (above) and element names (below). The number of profiles refers to how many different bonds of the same type are shown.

For the N-N-Rh-Cp* and N-N-Rh-Cl dihedrals, there are two distinct possibilities for which Nitrogen comes first, and which comes second. These two different dihedrals can be averaged. The advantage of the average is that values close to 180° signify that the Cp* or Cl ligand is in the symmetry plane vertical to the bpy plane. Offsets from 180° thus indicate asymmetrical distortions of the complex.

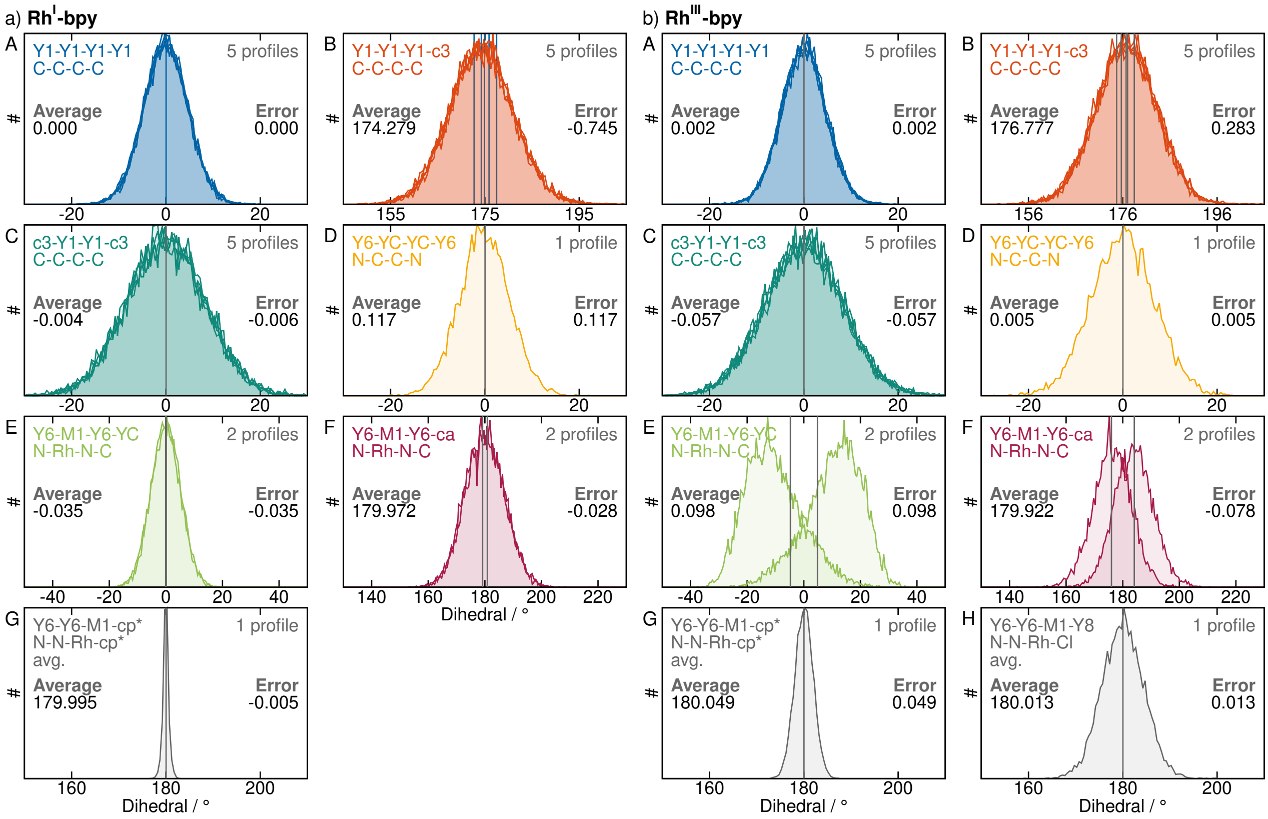

Figure S3: Characteristic dihedrals in a) Rh^{I}-bpy and b) Rh^{III}-bpy. The B3LYP reference values are shown as vertical lines. The bond definitions are given in terms of atom types according to Figure 2b (above) and element names (below). The number of profiles refers to how many different bonds of the same type are shown.

S3 Parametrization guide

In order to run a simulation of a given complex of the form [(bpy)Rh^{III}(Cp*)Cl]$^+$ or [(bpy)Rh^{I}(Cp*)] in Amber[1, 2], the following files are needed. There are some exceptions or other ways to do it, but I found this to be the most streamlined:

- an input.pdb file, which contains the initial structure of the entire complex
- mol2-files for the Rh center and for each ligand, which contain the atom type assignments and the atomic charges
- frcmod-files, which contain any force field-parameters not included in GAFF[3]/GAFF2[4]

I suggest the following protocol to generate these files. I outline the general procedure, but this is not a step-by-step tutorial for every program used, for instance MCPB or antechamber. However, there are tutorials for these out there, and in combination with this guide, it should be possible to generate the files.

First, generate an optimized equilibrium structure of the complex, for instance with B3LYP[5–7]/def2-SVP[8, 9] and Gaussian 16[10], and generate a pdb-file with this structure. From this pdb-file, generate mol2-files for the Rh center (with metalpdb2mol2.py, part of Amber) and all ligands (with antechamber, part of Amber). Carefully examine the atom types that antechamber assigns. Then, generate frcmod-files for each individual ligand using parmchk2 (part of Amber), which will assign additional terms if needed.

In order to get proper atomic charges, I recommend computing the electrostatic potential of the complex, for instance with B3LYP/def2-SVP and Gaussian 16. Using MCPB, the pdb and the mol2-files generated above, restricted electrostatic potential (RESP) charges can be fitted to this electrostatic potential. For this, step 1 in the MCPB routine can generate the input files MCPB needs, and step 3 performs the fitting, which will give new mol2-files for the metal center and each ligand. For the ligands, these charges need to be made symmetrical by hand, if they are not already. Furthermore, MCPB will assign custom atom types for the atoms involved in the metal bonds. If there are symmetry equivalent atoms, MCPB will still assign unique atom types, so they might need changing in order to have the same types. Make sure they have the same types as the ones in the manuscript. Additionally, the carbons in the 2,2' positions will need assignment of the YC atom type as described in the manuscript. These mol2-files, generated by MCPB and modified manually, are the ones to be used later with tleap.

Using the `rh1.frcmod` or `rh3.frcmod` files provided online with this paper (or the parameters listed above), the frcmod-files for the ligands generated with parmed, the mol2-files containing the RESP charges and atom type assignment and the initial structure in the pdb-file, you can generate the parameter-topology and input-coordinate files used by Amber's simulation engines with tleap (part of Amber).

S4 Comparison of RESP and NPA charges

In addition to the RESP charges presented in the manuscript, we computed natural charges, obtained from a natural population analysis (NPA), for Rh^{I}-bpy based on a natural bond orbital analysis[11–13] in Gaussian, using the B3LYP/def2-SVP setup described above. As evidenced by Figure S4, the differences between RESP and NPA charges are striking. The NPA charge for the Rh center is slightly positive, while it is ca. -0.7 in the case of RESP charges. For the organic ligands, the RESP charges are generally closer to 0, while the NPA charges cover a greater range, with the methyl carbon atoms in Cp* and the nitrogens in bpy exhibiting charges around -0.5.

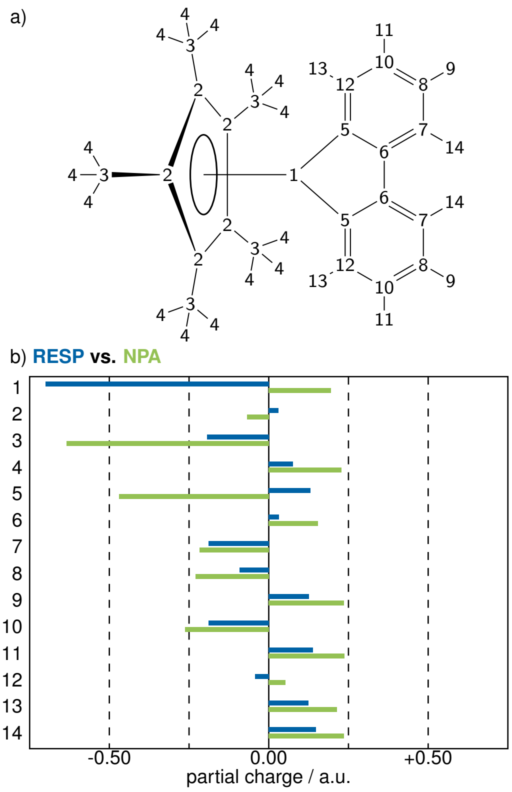

Figure S4: a) Numbering scheme for the atoms in Rh^{I}-bpy. b) RESP (blue) and NPA (green) charges in atomic units for Rh^{I}-bpy.

Table 1 shows the characteristic bond lenghts, angles and the dihedral that were already used for analysis in the manuscript, to compare the geometry between trajectories run with RESP and NPA charges. The results from the RESP charges are those presented in the manuscript. Even though the NPA charges are drastically different from the RESP charges, the different electrostatics merely influence the geometry of the complex. In general, the errors of the averages wrt. the B3LYP reference are larger for the NPA trajectories than for the RESP trajectories. However, the errors of all but one bond length are below 0.02 Å, and the largest error (for the M1-Y6 bond, which is the Rh-N bond) of -0.047 Å amounts to only ca. 2% of the total bond length, and can thus be considered insignificant. The errors for the angles and dihedrals are below 1°.

This analysis shows that for the parametrization and the resulting parameters presented in the manuscript, the charge method is of diminished importance, and our parameters are applicable in either case. Still, we want to briefly discuss which charge method is more suitable for our application,

Table 1: Averages (and errors wrt. the optimized B3LYP geometry) for selected bond lengths, angles and dihedrals of Rh^{I}-bpy with RESP and NPA charges. Distances in Å, angles and dihedrals in degrees.

Parameter	RESP	NPA
M1-Cp*	1.894 (0.003)	1.876 (-0.015)
M1-Y1	2.251 (0.000)	2.236 (-0.016)
M1-Y6	2.015 (0.004)	1.965 (-0.047)
Y1-Y1	1.433 (-0.006)	1.433 (-0.005)
Y6-YC	1.385 (0.001)	1.381 (-0.003)
YC-YC	1.444 (0.008)	1.443 (0.007)
Y6-M1-Cp*	140.5 (-0.1)	140.2 (-0.4)
Y6-M1-Y6	77.8 (-0.8)	79.0 (0.3)
M1-Y6-YC	117.7 (0.8)	117.8 (0.9)
Y1-Y1-Y1-c3	174.3 (-0.7)	174.1 (-0.9)

i.e., for the simulations of the interaction of Rh^{I}-bpy with the aqueous solvent. Thus, we computed the electrostatic potential of the complex with B3LYP/def2-SVP (as detailled above), shown in Figure S5. This electrostatic potential shows a negative potential near the Rhodium center as well as across the bpy ligand, though reduced in intensity of the latter. Thus, we judge it appropriate to assign a negative charge to the Rhodium, which allows to reproduce this negative potential. Granted, this negative charge might be overshot and probably needed to be compensated by the thus positively polarized nitrogens, but since the overarching goal was the simulation of the solvent interaction, we deem RESP charges adequate for this task.

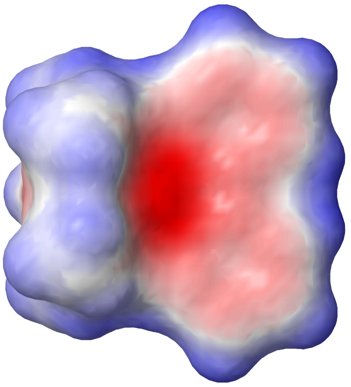

Figure S5: Electrostatic potential of Rh^{I}-bpy shown on the molecular surface. Red saturated areas show electrostatic potentials of -0.07 a.u., blue saturated areas +0.07 a.u.

References

(1) D. Case, H. Aktulga, K. Belfon, I. Ben-Shalom, J. Berryman, S. Brozell, D. Cerutti, T. Cheatham, G. Cisneros, V. Cruzeiro, T. Darden, N. Forouzesh, G. Giambasu, T. Giese, M. Gilson, H. Gohlke, A. Goetz, R. Harris, S. Izadi, S. Izmailov, K. Kasavajhala, M. Kaymak, E. King, A. Kovalenko, T. Kurtzman, T. Lee, P. Li, C. Lin, J. Liu, T. Luchko, R. Luo, M. Machado, V. Man, M. Manathunga, K. Merz, Y. Miao, O. Mikhailovskii, G. Monard, H. Nguyen, K. O'Hearn, A. Onufriev, F. Pan, S. Pantano, R. Qi, A. Rahnamoun, D. Roe, A. Roitberg, C. Sagui, S. Schott-Verdugo, A. Shajan, J. Shen, C. Simmerling, N. Skrynnikov, J. Smith, J. Swails, R. Walker, J. Wang, J. Wang, H. Wei, X. Wu, Y. Wu, Y. Xiong, Y. Xue, D. York, S. Zhao, Q. Zhu and P. Kollman, *Amber 2023*, San Fransisco, 2023.

(2) I. Case, D.A. Ben-Shalom, S. Brozell, D. Cerutti, T. Cheatham, V. Cruzeiro, T. Darden, R. Duke, M. Gilson, H. Gohlke, A. Goetz, D. Greene, R. Harris, N. Homeyer, Y. Huang, S. Izadi, A. Kovalenko, T. Kurtzman, T. Lee, S. LeGrand, P. Li, C. Lin, J. Liu, T. Luchko, R. Luo, D. J. Mermelstein, K. Merz, Y. Miao, G. Monard, C. Nguyen, H. Nguyen, I. Omelyan, A. Onufriev, F. Pan, R. Qi, D. Roe, A. Roitberg, C. Sagui, S. Schott-Verdugo, J. Shen, C. Simmerling, J. Smith, R. Salomon-Ferrer, J. Swails, R. Walker, J. Wang, H. Wei, R. Wolf, X. Wu, L. Xiao, D. York and P. Kollman, *Amber 2018*, San Fransisco, 2018.

(3) J. Wang, R. M. Wolf, J. W. Caldwell, P. A. Kollman and D. A. Case, *J. Comput. Chem.*, 2004, **25**, 1157–1174.

(4) J. Wang, W. Wang, P. A. Kollman and D. A. Case, *J. Mol. Graph. Model.*, 2006, **25**, 247–260.

(5) A. D. Becke, *J. Chem. Phys.*, 1993, **98**, 5648–5652.

(6) C. Lee, W. Yang and R. G. Parr, *Phys. Rev. B*, 1988, **37**, 785–789.

(7) P. J. Stephens, F. J. Devlin, C. F. Chabalowski and M. J. Frisch, *J. Phys. Chem.*, 1994, **98**, 11623–11627.

(8) F. Weigend and R. Ahlrichs, *Phys. Chem. Chem. Phys.*, 2005, **7**, 3297.

(9) F. Weigend, *Phys. Chem. Chem. Phys.*, 2006, **8**, 1057.

(10) M. J. Frisch, G. W. Trucks, H. B. Schlegel, G. E. Scuseria, M. A. Robb, J. R. Cheeseman, G. Scalmani, V. Barone, G. A. Petersson, H. Nakatsuji, X. Li, M. Caricato, A. V. Marenich, J. Bloino, B. G. Janesko, R. Gomperts, B. Mennucci, H. P. Hratchian, J. V. Ortiz, A. F. Izmaylov, J. L. Sonnenberg, D. Williams-Young, F. Ding, F. Lipparini, F. Egidi, J. Goings, B. Peng, A. Petrone, T. Henderson, D. Ranasinghe, V. G. Zakrzewski, J. Gao, N. Rega, G. Zheng, W. Liang, M. Hada, M. Ehara, K. Toyota, R. Fukuda, J. Hasegawa, M. Ishida, T. Nakajima, Y. Honda, O. Kitao, H. Nakai, T. Vreven, K. Throssell, J. A. Montgomery Jr., J. E. Peralta, F. Ogliaro, M. J. Bearpark, J. J. Heyd, E. N. Brothers, K. N. Kudin, V. N. Staroverov, T. A. Keith, R. Kobayashi, J. Normand, K. Raghavachari, A. P. Rendell, J. C. Burant, S. S. Iyengar, J. Tomasi, M. Cossi, J. M. Millam, M. Klene, C. Adamo, R. Cammi, J. W. Ochterski, R. L. Martin, K. Morokuma, O. Farkas, J. B. Foresman and D. J. Fox, *Gaussian16 Revision C.01*, 2016.

(11) J. P. Foster and F. Weinhold, *J. Am. Chem. Soc.*, 1980, **102**, 7211–7218.

(12) A. E. Reed, R. B. Weinstock and F. Weinhold, *J. Chem. Phys.*, 1985, **83**, 735–746.

(13) A. E. Reed, L. A. Curtiss and F. Weinhold, *Chem. Rev.*, 1988, **88**, 899–926.
